# Predictive values for different cancers and inflammatory bowel disease of 6 common abdominal symptoms among more than 1.9 million primary care patients in the UK: A cohort study

**DOI:** 10.1371/journal.pmed.1003708

**Published:** 2021-08-02

**Authors:** Annie Herbert, Meena Rafiq, Tra My Pham, Cristina Renzi, Gary A. Abel, Sarah Price, Willie Hamilton, Irene Petersen, Georgios Lyratzopoulos

**Affiliations:** 1 MRC Integrative Epidemiology Unit at University of Bristol, Bristol, United Kingdom; 2 Population Health Sciences, University of Bristol, Bristol, United Kingdom; 3 Epidemiology of Cancer Healthcare and Outcomes (ECHO) Research Group, Department of Behavioural Science and Health, University College London, London, United Kingdom; 4 MRC Clinical Trials Unit at UCL, London, United Kingdom; 5 University of Exeter Medical School, University of Exeter, Exeter, Devon, United Kingdom; 6 Department of Primary Care and Population Health, University College London, London, United Kingdom; 7 Department of Clinical Epidemiology, Aarhus University, Aarhus, Denmark; Harvard Medical School, UNITED STATES

## Abstract

**Background:**

The diagnostic assessment of abdominal symptoms in primary care presents a challenge. Evidence is needed about the positive predictive values (PPVs) of abdominal symptoms for different cancers and inflammatory bowel disease (IBD).

**Methods and findings:**

Using data from The Health Improvement Network (THIN) in the United Kingdom (2000–2017), we estimated the PPVs for diagnosis of (i) cancer (overall and for different cancer sites); (ii) IBD; and (iii) either cancer or IBD in the year post-consultation with each of 6 abdominal symptoms: dysphagia (*n* = 86,193 patients), abdominal bloating/distension (*n* = 100,856), change in bowel habit (*n* = 106,715), rectal bleeding (*n* = 235,094), dyspepsia (*n* = 517,326), and abdominal pain (*n* = 890,490). The median age ranged from 54 (abdominal pain) to 63 years (dysphagia and change in bowel habit); the ratio of women/men ranged from 50%:50% (rectal bleeding) to 73%:27% (abdominal bloating/distension). Across all studied symptoms, the risk of diagnosis of cancer and the risk of diagnosis of IBD were of similar magnitude, particularly in women, and younger men. Estimated PPVs were greatest for change in bowel habit in men (4.64% cancer and 2.82% IBD) and for rectal bleeding in women (2.39% cancer and 2.57% IBD) and lowest for dyspepsia (for cancer: 1.41% men and 1.03% women; for IBD: 0.89% men and 1.00% women). Considering PPVs for specific cancers, change in bowel habit and rectal bleeding had the highest PPVs for colon and rectal cancer; dysphagia for esophageal cancer; and abdominal bloating/distension (in women) for ovarian cancer. The highest PPVs of abdominal pain (either sex) and abdominal bloating/distension (men only) were for non-abdominal cancer sites. For the composite outcome of diagnosis of either cancer or IBD, PPVs of rectal bleeding exceeded the National Institute of Health and Care Excellence (NICE)-recommended specialist referral threshold of 3% in all age–sex strata, as did PPVs of abdominal pain, change in bowel habit, and dyspepsia, in those aged 60 years and over. Study limitations include reliance on accuracy and completeness of coding of symptoms and disease outcomes.

**Conclusions:**

Based on evidence from more than 1.9 million patients presenting in primary care, the findings provide estimated PPVs that could be used to guide specialist referral decisions, considering the PPVs of common abdominal symptoms for cancer alongside that for IBD and their composite outcome (cancer or IBD), taking into account the variable PPVs of different abdominal symptoms for different cancers sites. Jointly assessing the risk of cancer or IBD can better support decision-making and prompt diagnosis of both conditions, optimising specialist referrals or investigations, particularly in women.

## Abstract 2

Annie Herbert and co-workers study possible relevance of common abdominal symptoms for specialist referral in UK primary care.

## Author summary

**Why was this study done?:**

- The assessment of patients who present with abdominal symptoms in primary care is challenging.
- Abdominal symptoms are responsible for about 1 in 10 consultations but can relate to many underlying pathologies; predictive values for individual diseases are low.
- Prolonged intervals to diagnosis of cancer (often presenting with abdominal symptoms) and inflammatory bowel disease (IBD) are common and associated with worse patient outcomes.
- When cancer is suspected in patients with abdominal symptoms, several possible sites need to be considered, requiring different diagnostic modalities

**What did the researchers do and find?:**

- Using anonymous data from primary care records in the UK, we examined the predictive value of 6 common abdominal symptoms for cancer (overall and for specific cancer sites) and for IBD.
- Change in bowel habit and rectal bleeding had the highest positive predictive values (PPVs) for colon and rectal cancer; dysphagia for esophageal cancer; and abdominal bloating/distension (in women) for ovarian cancer.
- The highest PPVs for abdominal pain (either sex) and abdominal bloating/distension (men only) related to non-abdominal cancer sites.
- For diagnosis of either cancer or IBD, the predictive values of rectal bleeding exceeded guideline-recommended risk thresholds for specialist referral in all age–sex strata, as did the predictive values of abdominal pain, change in bowel habit, and dyspepsia, in those aged 60 years and over.

**What do these findings mean?:**

- Specialist referral decisions can be made considering the predictive values of common abdominal symptoms for cancer alongside that for IBD and the composite outcome of either cancer or IBD.
- Jointly assessing the risk of cancer or IBD can better support decision-making and prompt diagnosis of both conditions, enabling specialist referrals or investigations, particularly of women.
- Investigation strategies and use of diagnostic modalities can be guided by the stratification of risk of different abdominal symptoms for different cancers sites.

## Introduction

The assessment of new abdominal symptoms in primary care is challenging. About 1 in 10 consulting patients present with at least 1 abdominal symptom (e.g., abdominal pain, change in bowel habit, and bloating/distension) [1]. This high relative frequency of abdominal symptom presentations is combined with a large range of possible underlying pathologies. In many cancers, including those typically presenting with abdominal symptoms, prolonged intervals between the first presentation with relevant symptoms and diagnosis are common and associated with poorer clinical and patient-reported outcomes [2,3]. Substantial delays in the diagnosis of inflammatory bowel disease (IBD) have also been reported [4]. Further, when cancer is suspected in patients presenting with abdominal symptoms, several cancer sites need to be considered, although they often have different principal diagnostic modalities (e.g., colorectal cancer: colonoscopy, pancreatic cancer: abdominal computerised tomography, and ovarian cancer: transvaginal ultrasound) [1,5].

For symptoms associated with relatively high predictive values for cancer (i.e., >3%), current National Institute of Health and Care Excellence (NICE) clinical practice guidelines recommend that general practitioners (GPs) investigate or refer patients for urgent specialist assessment through fast track, “two-week-wait” pathways [6]. Prior evidence examining the predictive value of abdominal symptoms tends to focus on individual cancer sites considered in isolation, although the same symptom may be associated with different cancer sites [7]. Further, the spectrum of tumours in patients presenting with abdominal symptoms includes cancers of organs adjacent to the abdominal cavity and of non-abdominal organs [5]. A single prior study that considered the positive predictive values (PPVs) of abdominal symptoms for a combined outcome of either cancer or IBD was confined to patients under age 50 years [8], although most patients with cancer are diagnosed in older ages. Both (abdominal organ) cancers and IBD often present with similar symptoms, both require prompt diagnosis, and both involve referrals to gastroenterology and colonoscopy services.

Against this background and given that the diagnostic strategies to investigate suspected cancer or IBD in patients presenting with abdominal symptoms typically involve specialist assessment and endoscopic investigation, we aimed to estimate the predictive values of common abdominal symptom presentations to primary care for (i) cancer; (ii) IBD; and (iii) the composite outcome of cancer or IBD. We focused in particular on predictive values exceeding the 3% threshold used by NICE to determine the need for specialist assessment or investigation.

## Methods

### Study design

We carried out a retrospective population-based cohort study using routinely collected electronic health record data from The Health Improvement Network (THIN) primary care database between 2000 and 2017. These data include information on patient and consultation characteristics (including information on symptoms and diagnoses recorded using Read codes) [9] for patients registered with UK GP practices contributing data to THIN. This includes 742 practices, covering approximately 6.2% of the UK population, and considered to be reasonably representative of it [10]. Previous research has demonstrated the validity of this source (THIN) for ascertaining new diagnoses of cancer [11–13]. Comprehensive Read symptom code lists developed previously were used to identify the 6 abdominal symptoms of interest (abdominal bloating/distension, abdominal pain, change in bowel habit, dyspepsia, dysphagia, and rectal bleeding) [14,15].

We derived 6 abdominal symptom cohorts corresponding to the above 6 abdominal symptoms of interest. How the 6 symptom cohorts were derived is shown in a flowchart (**S1 Diagram**), from the initial sample of patients in THIN in 2000 to 2017 and excluding patients in a stepwise process to meet the inclusion criteria.

The inclusion criteria were that patients had to have at least 1 consultation record that included the abdominal symptom of interest, at age 30 to 99 years, during 2001 to 2016, where that consultation record also met the following criteria: (i) any consultations in both the previous and the following year to the consultation of interest would be captured; (ii) both the consultation record of interest and any consultations in the previous or the following year would be of acceptable recording standards (i.e., occurring during dates where the practice had passed validation checks and achieved current quality standards [16,17], and the patient had been registered for at least 6 months); (iii) no records of consultations in the year prior to the consultation of interest indicated diagnosis of either cancer or IBD.

Within each cohort, for patients with more than 1 eligible consultation for the symptom of interest, we randomly selected one to be the index consultation. This was to represent better any new presentations of symptoms in everyday practice (where “new” here means first consultation for that symptom in at least a year). Patients could have been included in more than 1 of the 6 symptom cohorts, and, in such cases, the dates of index consultations for different symptoms could be different for the same patient. The STrengthening the Reporting of OBservational studies in Epidemiology (STROBE) reporting guidelines checklist for cohort studies is included as a Supporting information file (**S1 Text**).

### Exposures, outcomes, and other covariates

Exposures comprised each of the 6 abdominal symptoms. The 3 principal outcomes were (i) diagnosis of cancer; (ii) diagnosis of IBD; (iii) diagnosis of either cancer or IBD, in the year following the index consultation. We considered cancer overall (i.e., any cancer site), but also by specific site. Cancer sites examined included common cancers of abdominal or adjacent organs deemed most relevant to the studied 6 symptoms (i.e., colon, rectal, esophageal, stomach, pancreatic, ovarian, uterine, and renal cancer). For completeness, we also considered sarcoma and lymphoma as cancers with possible intra-abdominal manifestations (therefore possibly presenting with abdominal symptom) and a heterogeneous “other cancer” group (i.e., comprising cancers whose presenting symptoms typically exclude the studied abdominal symptoms, including bladder, breast, cervical, laryngeal, lung, melanoma, multiple myeloma, prostate, testicular, thyroid, vaginal, and vulval cancer). Both cancer and IBD were identified using Read code lists that have been previously developed [15]. Other covariates were sex and age group (30 to 39, 40 to 49, 50 to 59, 60 to 69, 70 to 79, and 80+ years) at the time of index consultation.

### Statistical analysis

We calculated the PPVs of cancer, IBD, and either cancer or IBD, respectively, in the year following the index consultation (equal to the proportions of patients who were diagnosed with cancer, IBD, and either cancer or IBD). We first estimated PPVs for each of the symptoms considered in isolation and then in combination with each of the other 5 abdominal symptoms if they had been recorded in consultations in the previous year, up to and including during the index consultation. To achieve a greater understanding of the risks of different types of cancer, we additionally calculated PPVs for each of the cancer sites described above.

Concordant with prior evidence and guideline recommendations, we estimated all PPVs for men and women separately [6] and then by age group [18–22]. To avoid presenting small numbers and PPVs with wide confidence intervals (CIs), for cancer-specific PPVs, we present PPVs by age group and sex for colon, rectal, esophageal, and non-abdominal cancer only. The preliminary analysis plan is included as a Supporting information file (**S2 Text**). These analyses were carried out as planned, with 1 set of post hoc analyses: to provide additional context of the 12-month risk of cancer and IBD associated with presentation with each of the studied symptoms, we estimated the number of cases of cancer and IBD that would have been expected in each symptom cohort given the age-specific incidence of cancer, and IBD, using population-based sources for incidence and population estimates [23–25].

We analysed the data using Stata version 15.1 [26].

### Approvals

The data provider (IQVIA) obtained overall ethical approval for the use of THIN in scientific research from the South East Medical Research Ethics Committee (MREC/03/01/073), and this study was further approved by the THIN Scientific Review Committee (18THIN069).

## Results

### Study population and patient characteristics

During 2000 to 2017, 16,421,201 patients consulted at least once (for any reason) in the 742 general practices included in THIN (**S1 Diagram**). After exclusions, the number of patients with at least 1 consultation with a relevant symptom during 2001 to 2016 was 102,785 for abdominal bloating/distension, 909,451 for abdominal pain, 108,698 for change in bowel habit, 528,428 for dyspepsia, 87,971 for dysphagia, and 240,253 for rectal bleeding (**S1 Diagram**). Across the 6 symptom cohorts, between 86,193 and 890,490 patients had at least 1 consultation for the relevant symptom, which was not preceded by consultations for the same symptom, cancer, or IBD in the previous year.

**Table 1** describes the composition of the 6 symptom cohorts by patient characteristic. Except for rectal bleeding, there was a preponderance of women across the cohorts, ranging from 118,371 (50%) for rectal bleeding to 73,583 (73%) for abdominal bloating/distension. The median age at the time of the index consultation ranged from 52 years in the abdominal pain cohort to 63 years in the change in bowel habit and dysphagia cohorts. For abdominal pain, change in bowel habit, dyspepsia, dysphagia, and rectal bleeding, a large majority (87% to 99%) of patients had a single abdominal symptom recorded at their index consultation, and, typically (75% to 92%), without any previous consultations for the other studied abdominal symptoms in the previous year. A contrasting pattern was observed for abdominal bloating/distension, where 66% of all patients had at least another abdominal symptom recorded at their index consultation, and 75% had at least another abdominal symptom recorded either at the index consultation or in the previous year (most frequently for dyspepsia).

**Table 1:** Characteristics of patients per studied symptom cohort at time of index consultation, *n* (%).

Demographic and clinical variables	Abdominal bloating/distension *n*/(%)		Abdominal pain *n*/(%)		Change in bowel habit *n*/(%)		Dyspepsia *n*/(%)		Dysphagia *n*/(%)		Rectal bleeding *n*/(%)	
**Total**	100,856	(100.0)	890,490	(100.0)	106,715	(100.0)	517,326	(100.0)	86,193	(100.0)	235,094	(100.0)
**Sex**												
Female	73,583	(73.0)	561,373	(63.0)	61,476	(57.6)	300,053	(58.0)	48,759	(56.6)	118,371	(50.4)
**Age (years)**												
Median (IQR)	53	(43–66)	52	(41–66)	63	(52–73)	56	(44–68)	63	(51–75)	56	(46–69)
**Age group (years)**												
30–39	17,709	(17.6)	198,916	(22.3)	6,666	(6.2)	79,425	(15.4)	6,988	(8.1)	39,042	(16.6)
40–49	23,915	(23.7)	198,502	(22.3)	14,596	(13.7)	105,389	(20.4)	12,705	(14.7)	47,676	(20.3)
50–59	21,124	(20.9)	173,876	(19.5)	23,103	(21.6)	108,031	(20.9)	16,496	(19.1)	48,879	(20.8)
60–69	18,029	(17.9)	152,039	(17.1)	26,165	(24.5)	108,086	(20.9)	18,372	(21.3)	44,231	(18.8)
70–79	13,354	(13.2)	109,848	(12.3)	23,489	(22.0)	78,729	(15.2)	17,566	(20.4)	34,472	(14.7)
80+	6,725	(6.7)	57,309	(6.4)	12,696	(11.9)	37,666	(7.3)	14,066	(16.3)	20,794	(8.8)
**Number of abdominal symptoms at index** ^*^												
1	31,948	(31.7)	881,099	(98.9)	102,613	(96.2)	452,348	(87.4)	84,259	(97.8)	229,746	(97.7)
2	66,154	(65.6)	8,156	(0.9)	3,552	(3.3)	62,917	(12.2)	1,812	(2.1)	4,942	(2.1)
3+	2,754	(2.7)	1,235	(0.1)	550	(0.5)	2,061	(0.4)	122	(0.1)	406	(0.2)
**Other abdominal symptoms** ^**^												
None	24,899	(24.7)	819,828	(92.1)	86,118	(80.7)	389,398	(75.3)	71,515	(83.0)	202,794	(86.3)
Any	75,957	(75.3)	70,662	(7.9)	20,597	(19.3)	127,928	(24.7)	14,678	(17.0)	32,300	(13.7)
Abdominal bloating/distension			10,572	(1.2)	1,715	(1.6)	60,690	(11.7)	852	(1.0)	2,029	(0.9)
Abdominal pain	16,650	(16.5)			12,701	(11.9)	59,594	(11.5)	7,463	(8.7)	20,537	(8.7)
Change in bowel habit	1,418	(1.4)	7,399	(0.8)			4,163	(0.8)	714	(0.8)	3,056	(1.3)
Dyspepsia	70,679	(70.1)	44,309	(5.0)	6,068	(5.7)			6,753	(7.8)	10,286	(4.4)
Dysphagia	759	(0.8)	4,552	(0.5)	793	(0.7)	9,356	(1.8)			1,361	(0.6)
Rectal bleeding	1,941	(1.9)	13,565	(1.5)	3,307	(3.1)	8,022	(1.6)	1,336	(1.6)		

* Number of symptoms recorded simultaneously on index consultation date.

** Symptoms recorded in the same consultations or up to a year prior. Note that patients can have multiple abdominal symptoms in the previous year, and, therefore, %s may not add to 100.

### Positive predictive values of the studied abdominal symptoms

The number of new diagnoses of cancer and new diagnoses of IBD observed in each cohort, together with PPVs for cancer, are presented in **Table 2**. Overall, the number of patients diagnosed with any cancer and those diagnosed with IBD was of similar magnitude, although in men, PPVs for cancer were higher than those for IBD for all abdominal symptoms (e.g., abdominal pain: 1.77% versus 1.17%; **Table 2**). In contrast, in women, the risk of cancer and the risk of IBD tended to be similar; further, for change in bowel habit and for rectal bleeding, the risk of IBD exceeded that for cancer.

**Table 2:** Number of incident cases and PPVs (95% CIs) for cancer and for IBD in the year following each studied abdominal symptom, considering each symptom regardless of other symptoms, or in pairwise combinations with the other examined symptoms*.

Symptom cohort Pairwise symptom combinations (recorded in the same consultation or up to a year prior)	Men						Women					
	Cancer			IBD			Cancer			IBD		
	*n*	PPV (%)	(95% CI)	*n*	PPV (%)	(95% CI)	*n*	PPV (%)	(95% CI)	*n*	PPV (%)	(95% CI)
**Abdominal bloating/distension**	450	1.65	(1.50, 1.80)	296	1.09	(0.96, 1.21)	982	1.33	(1.25, 1.42)	733	1.00	(0.92, 1.07)
Combined with the following:												
Abdominal pain	110	2.53	(2.06, 2.99)	71	1.63	(1.25, 2.01)	207	1.68	(1.46, 1.91)	196	1.59	(1.37, 1.82)
Change in bowel habit	10	2.21	(0.85, 3.56)	7	1.55	(0.41, 2.68)	24	2.49	(1.50, 3.47)	14	1.45	(0.70, 2.21)
Dyspepsia	271	1.45	(1.27, 1.62)	186	0.99	(0.85, 1.13)	547	1.05	(0.97, 1.14)	485	0.93	(0.85, 1.02)
Dysphagia	9	3.91	(1.41, 6.42)	<5	<2.17		6	1.13	(0.23, 2.04)	8	1.51	(0.47, 2.55)
Rectal bleeding	16	2.43	(1.25, 3.60)	20	3.03	(1.73, 4.34)	21	1.64	(0.94, 2.33)	33	2.57	(1.71, 3.44)
**Abdominal pain**	5,841	1.77	(1.73, 1.82)	3,842	1.17	(1.13, 1.20)	6,749	1.20	(1.17, 1.23)	6,544	1.17	(1.14, 1.19)
Combined with the following:												
Abdominal bloating/distension	81	2.86	(2.24, 3.47)	34	1.20	(0.80, 1.60)	144	1.86	(1.56, 2.16)	104	1.34	(1.09, 1.60)
Change in bowel habit	130	4.64	(3.86, 5.42)	84	3.00	(2.37, 3.63)	123	2.68	(2.21, 3.14)	120	2.61	(2.15, 3.07)
Dyspepsia	337	2.02	(1.81, 2.23)	208	1.25	(1.08, 1.41)	426	1.54	(1.40, 1.69)	359	1.30	(1.17, 1.43)
Dysphagia	52	3.15	(2.31, 4.00)	26	1.58	(0.98, 2.18)	65	2.24	(1.70, 2.78)	56	1.93	(1.43, 2.43)
Rectal bleeding	176	3.02	(2.58, 3.46)	215	3.69	(3.21, 4.18)	157	2.03	(1.71, 2.34)	227	2.93	(2.56, 3.31)
**Change in bowel habit**	2,101	4.64	(4.45, 4.84)	1,278	2.82	(2.67, 2.98)	1,471	2.39	(2.27, 2.51)	1,581	2.57	(2.45, 2.70)
Combined with the following:												
Abdominal bloating/distension	16	3.29	(1.71, 4.88)	11	2.26	(0.94, 3.59)	26	2.12	(1.31, 2.92)	29	2.36	(1.51, 3.21)
Abdominal pain	211	4.78	(4.15, 5.41)	111	2.51	(2.05, 2.97)	174	2.10	(1.79, 2.41)	209	2.52	(2.19, 2.86)
Dyspepsia	66	2.95	(2.25, 3.65)	52	2.32	(1.70, 2.95)	73	1.91	(1.47, 2.34)	112	2.92	(2.39, 3.46)
Dysphagia	14	4.46	(2.18, 6.74)	10	3.18	(1.24, 5.13)	9	1.88	(0.66, 3.09)	14	2.92	(1.41, 4.43)
Rectal bleeding	121	8.46	(7.01, 9.90)	93	6.50	(5.22, 7.78)	98	5.22	(4.22, 6.23)	92	4.90	(3.93, 5.88)
**Dyspepsia (any)**	3,072	1.41	(1.36, 1.46)	1,941	0.89	(0.85, 0.93)	3,091	1.03	(0.99, 1.07)	2,998	1.00	(0.96, 1.03)
Combined with the following:												
Abdominal bloating/distension	232	1.48	(1.29, 1.67)	164	1.04	(0.89, 1.20)	495	1.10	(1.00, 1.20)	414	0.92	(0.83, 1.01)
Abdominal pain	363	1.67	(1.50, 1.84)	272	1.25	(1.10, 1.40)	439	1.16	(1.05, 1.27)	492	1.30	(1.19, 1.41)
Change in bowel habit	35	2.16	(1.46, 2.87)	29	1.79	(1.15, 2.44)	29	1.14	(0.73, 1.55)	56	2.20	(1.63, 2.77)
Dysphagia	106	2.31	(1.88, 2.75)	37	0.81	(0.55, 1.07)	83	1.74	(1.37, 2.11)	53	1.11	(0.81, 1.41)
Rectal bleeding	63	1.63	(1.23, 2.03)	78	2.02	(1.58, 2.47)	72	1.73	(1.33, 2.13)	89	2.14	(1.70, 2.58)
**Dysphagia**	1,601	4.28	(4.07, 4.48)	282	0.75	(0.67, 0.84)	1,041	2.13	(2.01, 2.26)	441	0.90	(0.82, 0.99)
Combined with the following:												
Abdominal bloating/distension	5	2.25	(0.30, 4.20)	<5	<2.32		10	1.59	(0.61, 2.56)	<5	<0.78	
Abdominal pain	123	5.10	(4.22, 5.98)	30	1.24	(0.80, 1.69)	101	2.00	(1.61, 2.39)	71	1.41	(1.08, 1.73)
Change in bowel habit	15	5.36	(2.72, 7.99)	<5	<1.83		11	2.53	(1.06, 4.01)	5	1.15	(0.15, 2.16)
Dyspepsia	149	5.69	(4.80, 6.57)	18	0.69	(0.37, 1.00)	98	2.37	(1.91, 2.84)	35	0.85	(0.57, 1.13)
Rectal bleeding	25	4.07	(2.50, 5.63)	11	1.79	(0.74, 2.84)	15	2.08	(1.04, 3.12)	11	1.53	(0.63, 2.42)
**Rectal bleeding**	3,730	3.20	(3.09, 3.30)	3,080	2.64	(2.55, 2.73)	2,911	2.46	(2.37, 2.55)	3,271	2.76	(2.67, 2.86)
Combined with the following:												
Abdominal bloating/distension	18	2.97	(1.62, 4.32)	22	3.63	(2.14, 5.12)	29	2.04	(1.30, 2.77)	40	2.81	(1.95, 3.67)
Abdominal pain	225	2.94	(2.56, 3.32)	192	2.51	(2.16, 2.86)	255	1.98	(1.74, 2.22)	367	2.85	(2.56, 3.14)
Change in bowel habit	94	7.11	(5.73, 8.50)	82	6.20	(4.90, 7.50)	98	5.65	(4.56, 6.74)	108	6.23	(5.09, 7.37)
Dyspepsia	113	2.45	(2.01, 2.90)	107	2.32	(1.89, 2.76)	102	1.80	(1.45, 2.14)	151	2.66	(2.24, 3.08)
Dysphagia	13	2.18	(1.01, 3.36)	12	2.02	(0.89, 3.15)	21	2.74	(1.59, 3.90)	25	3.26	(2.01, 4.52)

Counts of cancer <5 or of IBD <5 in Table 2 are not presented, in compliance with reporting standards for minimising disclosivity risk.

* The number of patients with both cancer and IBD in the year following the index abdominal symptom consultation (regardless of prior symptoms) ranged from 7 (men with abdominal bloating/distension or dysphagia) to 95 (women with abdominal pain). See Table 3 for numbers and PPVs for the composite outcome of either cancer or IBD.

CI, confidence interval; IBD, inflammatory bowel disease; PPV, positive predictive value.

Across the 6 symptom cohorts, 1.41% (95% CI: 1.36% to 1.46%) to 4.64% (4.45% to 4.84%) of men and 1.03% (0.99% to 1.07%) to 2.46% (2.37% to 2.55%) of women were diagnosed with cancer in the year following their index consultation. For each studied abdominal symptom, PPVs for cancer were higher in men than in women. Change in bowel habit, dysphagia, and rectal bleeding were the 3 symptoms with relatively higher PPVs for cancer (men: 4.68%, 4.28%, and 3.20%, respectively, and women: 2.57%, 2.13%, and 2.46%, respectively).

Risks (for both cancer and for IBD) were influenced by whether a symptom was recorded “in combination” with other abdominal symptoms (either at the same consultation or during another consultation in the preceding 12 months; **Table 2**). For example, abdominal bloating/distension in men had a PPV for cancer of 1.65% (95% CI: 1.50% to 1.80%), increasing to 2.53% (2.06% to 2.99%) when combined with abdominal pain. In both sexes, PPVs increased notably if the index consultation was combined with change in bowel habit (among those presenting with any of the other 5 symptoms) or with rectal bleeding (among those presenting with abdominal bloating/distension and change in bowel habit). Consequently, the highest PPVs were seen among those presenting with change in bowel habit combined with rectal bleeding or vice versa; in these strata, the risk of cancer or IBD ranged between 5% and 8% in the year following the index consultation. Among the patients subsequently diagnosed with cancer, the percentage of those with more than 1 symptom recorded in the same consultation or the following year was greatest for abdominal bloating/distension (56%) and dyspepsia (13%), with smaller respective percentages for change in bowel habit (5%) and rectal bleeding, dysphagia, and abdominal pain (2% for all 3).

Considering the composite outcome of either cancer or IBD across the 6 symptom cohorts, PPVs ranged from 2.29% (2.23% to 2.36%) to 7.41% (7.17% to 7.65%) in men and from 2.02% (95% CI: 1.97% to 2.07%) to 5.18% (5.05% to 5.30%) in women (**Table 3**). Both men and women presenting with change in bowel habit, dysphagia, or rectal bleeding had PPVs for either cancer or IBD exceeding 3%.

**Table 3:** Numbers of incident cases and PPVs (95% CIs) for the composite outcome of either cancer or IBD in the year following each studied abdominal symptom.

	Men			Women		
	Number diagnosed with either cancer or IBD	PPV (%)	(95% CI)	Number diagnosed with either cancer or IBD	PPV (%)	(95% CI)
**Abdominal bloating/distension**	739	2.71	(2.52, 2.90)	1,707	2.32	(2.21, 2.43)
Combined with the following:						
Abdominal pain	180	4.13	(3.54, 4.72)	401	3.26	(2.95, 3.58)
Change in bowel habit	17	3.75	(2.00, 5.50)	37	3.83	(2.62, 5.05)
Dyspepsia	453	2.42	(2.20, 2.64)	1,028	1.98	(1.86, 2.10)
Dysphagia	<14	<6.09		14	2.65	(1.28, 4.01)
Rectal bleeding	35	5.31	(3.60, 7.02)	54	4.21	(3.11, 5.31)
**Abdominal pain**	9,607	2.92	(2.86, 2.98)	13,198	2.35	(2.31, 2.39)
Combined with the following:						
Abdominal bloating/distension	115	4.05	(3.33, 4.78)	247	3.19	(2.80, 3.59)
Change in bowel habit	212	7.57	(6.59, 8.55)	241	5.24	(4.60, 5.89)
Dyspepsia	542	3.25	(2.98, 3.52)	780	2.82	(2.63, 3.02)
Dysphagia	78	4.73	(3.71, 5.75)	120	4.13	(3.41, 4.86)
Rectal bleeding	390	6.70	(6.06, 7.34)	381	4.92	(4.44, 5.40)
**Change in bowel habit**	3,353	7.41	(7.17, 7.65)	3,015	4.90	(4.73, 5.08)
Combined with the following:						
Abdominal bloating/distension	27	5.56	(3.52, 7.59)	53	4.31	(3.18, 5.45)
Abdominal pain	321	7.27	(6.50, 8.03)	379	4.58	(4.13, 5.03)
Dyspepsia	118	5.27	(4.35, 6.20)	179	4.67	(4.01, 5.34)
Dysphagia	23	7.32	(4.44, 10.21)	22	4.59	(2.72, 6.47)
Rectal bleeding	212	14.81	(12.97, 16.66)	188	10.02	(8.66, 11.38)
**Dyspepsia**	4,983	2.29	(2.23, 2.36)	6,063	2.02	(1.97, 2.07)
Combined with the following:						
Abdominal bloating/distension	393	2.50	(2.26, 2.75)	905	2.01	(1.88, 2.14)
Abdominal pain	632	2.90	(2.68, 3.13)	927	2.45	(2.29, 2.61)
Change in bowel habit	63	3.90	(2.95, 4.84)	85	3.34	(2.64, 4.04)
Dysphagia	142	3.10	(2.60, 3.60)	136	2.85	(2.38, 3.32)
Rectal bleeding	141	3.65	(3.06, 4.25)	160	3.84	(3.26, 4.43)
**Dysphagia**	1,874	5.01	(4.79, 5.23)	1,474	3.02	(2.87, 3.18)
Combined with the following:						
Abdominal bloating/distension	<10	<4.50		<15	<2.38	
Abdominal pain	150	6.22	(5.26, 7.19)	171	3.38	(2.89, 3.88)
Change in bowel habit	<20	<7.14		16	3.69	(1.91, 5.46)
Dyspepsia	167	6.37	(5.44, 7.31)	132	3.19	(2.66, 3.73)
Rectal bleeding	36	5.85	(4.00, 7.71)	26	3.61	(2.25, 4.97)
**Rectal bleeding**	6,768	5.80	(5.66, 5.93)	6,128	5.18	(5.05, 5.30)
Combined with the following:						
Abdominal bloating/distension	39	6.44	(4.48, 8.39)	69	4.85	(3.73, 5.96)
Abdominal pain	414	5.41	(4.90, 5.92)	614	4.77	(4.40, 5.13)
Change in bowel habit	176	13.31	(11.48, 15.14)	204	11.76	(10.25, 13.28)
Dyspepsia	218	4.74	(4.12, 5.35)	251	4.42	(3.88, 4.95)
Dysphagia	25	4.20	(2.59, 5.81)	46	6.01	(4.32, 7.69)

Total counts of cancer or IBD, where counts of cancer <5 or of IBD <5 in Table 2, are not presented here (in Table 3) in compliance with reporting standards for minimising disclosivity risk.

CI, confidence interval; IBD, inflammatory bowel disease; PPV, positive predictive value.

**Fig 1:**
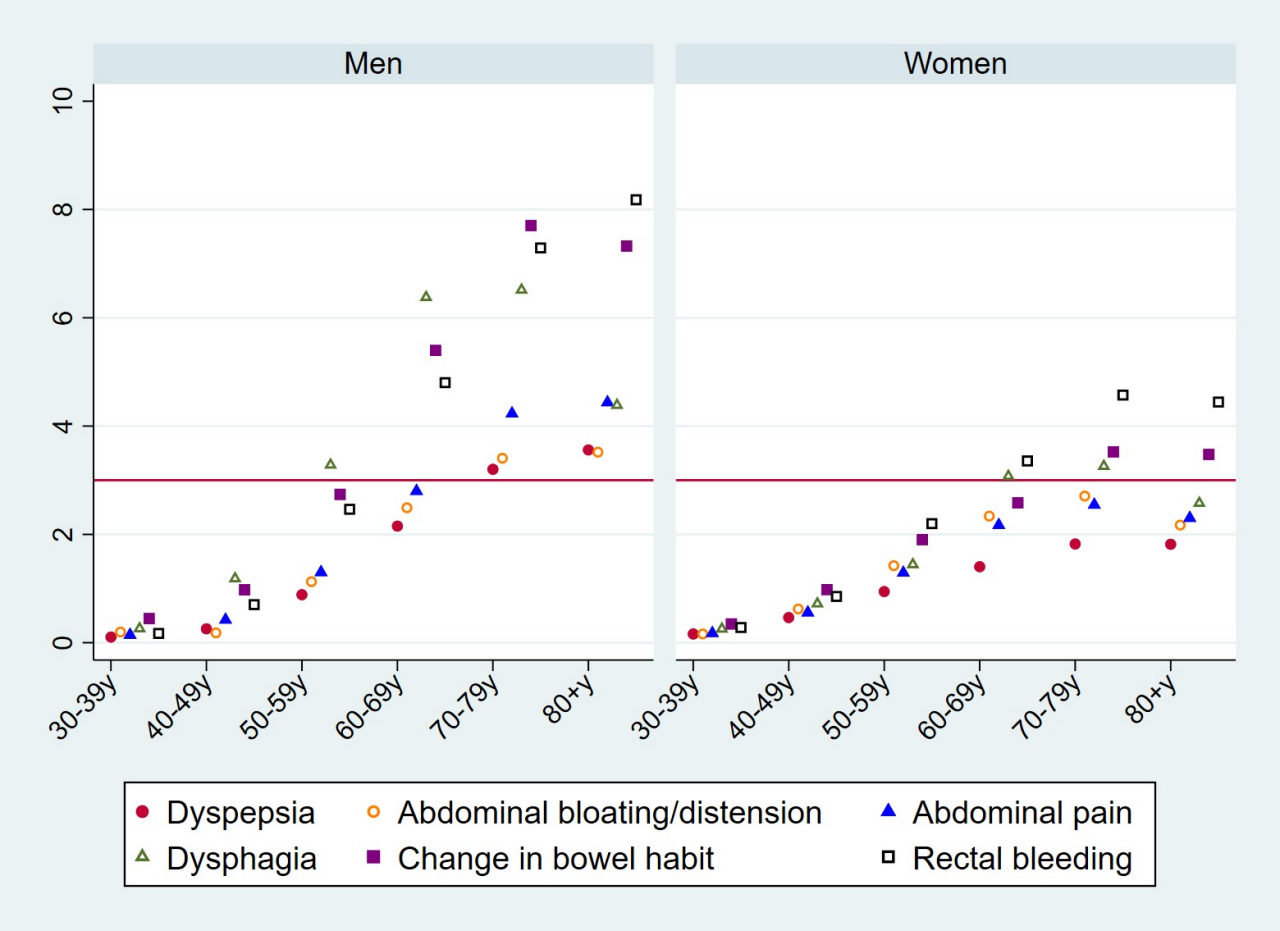
PPVs (%, *y* axis) for cancer in the year post-presentation, per studied symptom, by sex (left/right panels) and age group (*x* axis). The red horizontal line represents the NICE 3% or greater PPV threshold for urgent (two-week-wait) referral for suspected cancer. Exact values from this figure are provided in S1 Table. PPV, positive predictive value.

PPVs for cancer by sex–age group are shown in **Fig 1** (see **S1 Table** for exact values). Those increased with age, particularly in men presenting with change in bowel habit, dysphagia, and rectal bleeding. Although PPVs for cancer were similar between men and women in younger age groups, PPVs among those aged 60 years or over were higher in men.

**Fig 2:**
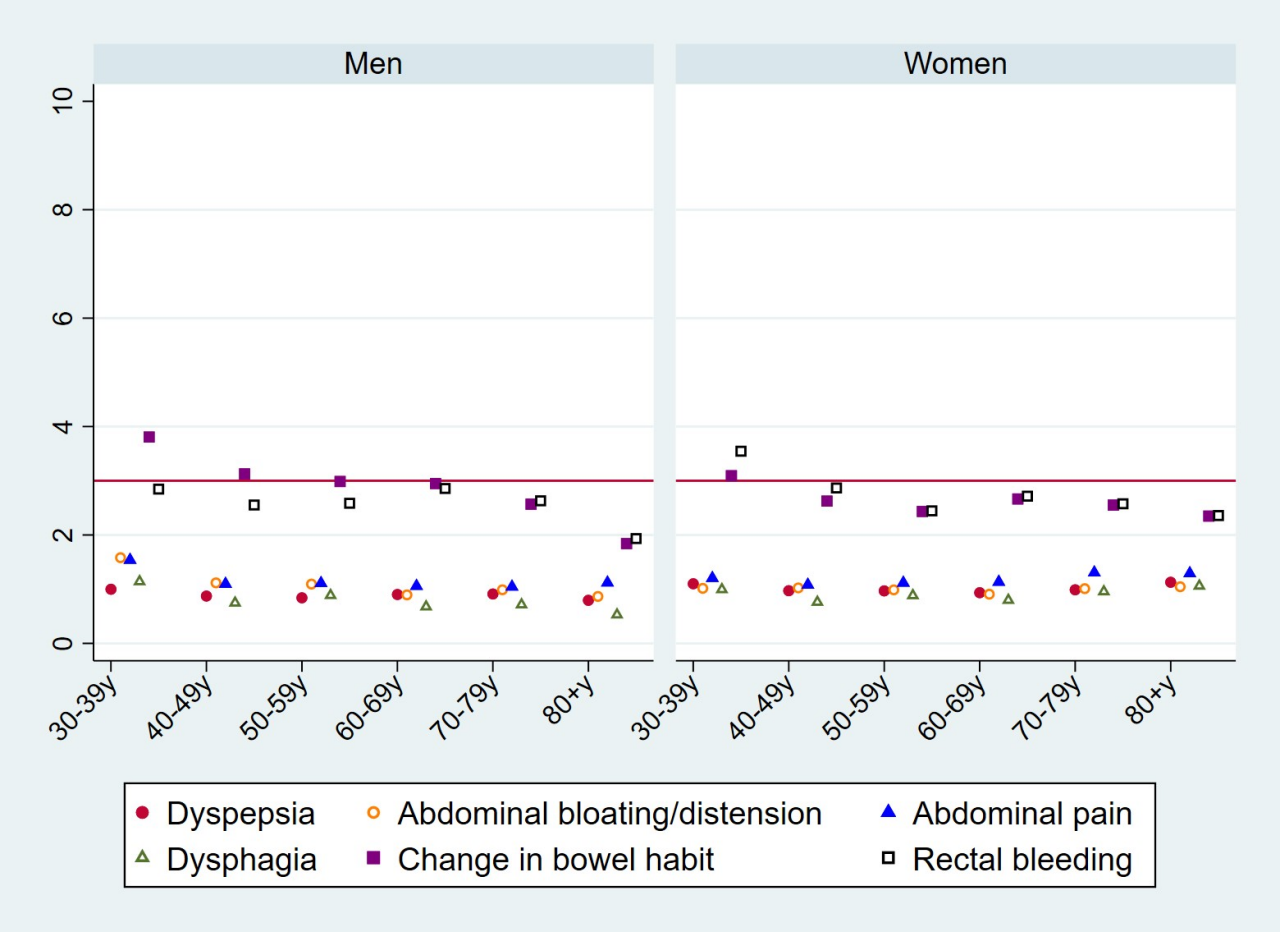
PPVs (%, *y* axis) for IBD in the year post-presentation, per studied symptom, by sex (left/right panels) and age group (*x* axis). The red horizontal line represents the NICE 3% or greater PPV threshold for urgent (two-week-wait) referral for suspected cancer. Exact values are provided in S1 Table. IBD, inflammatory bowel disease; PPV, positive predictive value.

In contrast to PPVs for cancer, those for IBD remained similar across age groups (**Fig 2**; see **S1 Table** for exact values) and exceeded PPVs for cancer in younger age groups (30 to 39 and 40 to 49 years; **Figs 1 and 2**).

**Fig 3:**
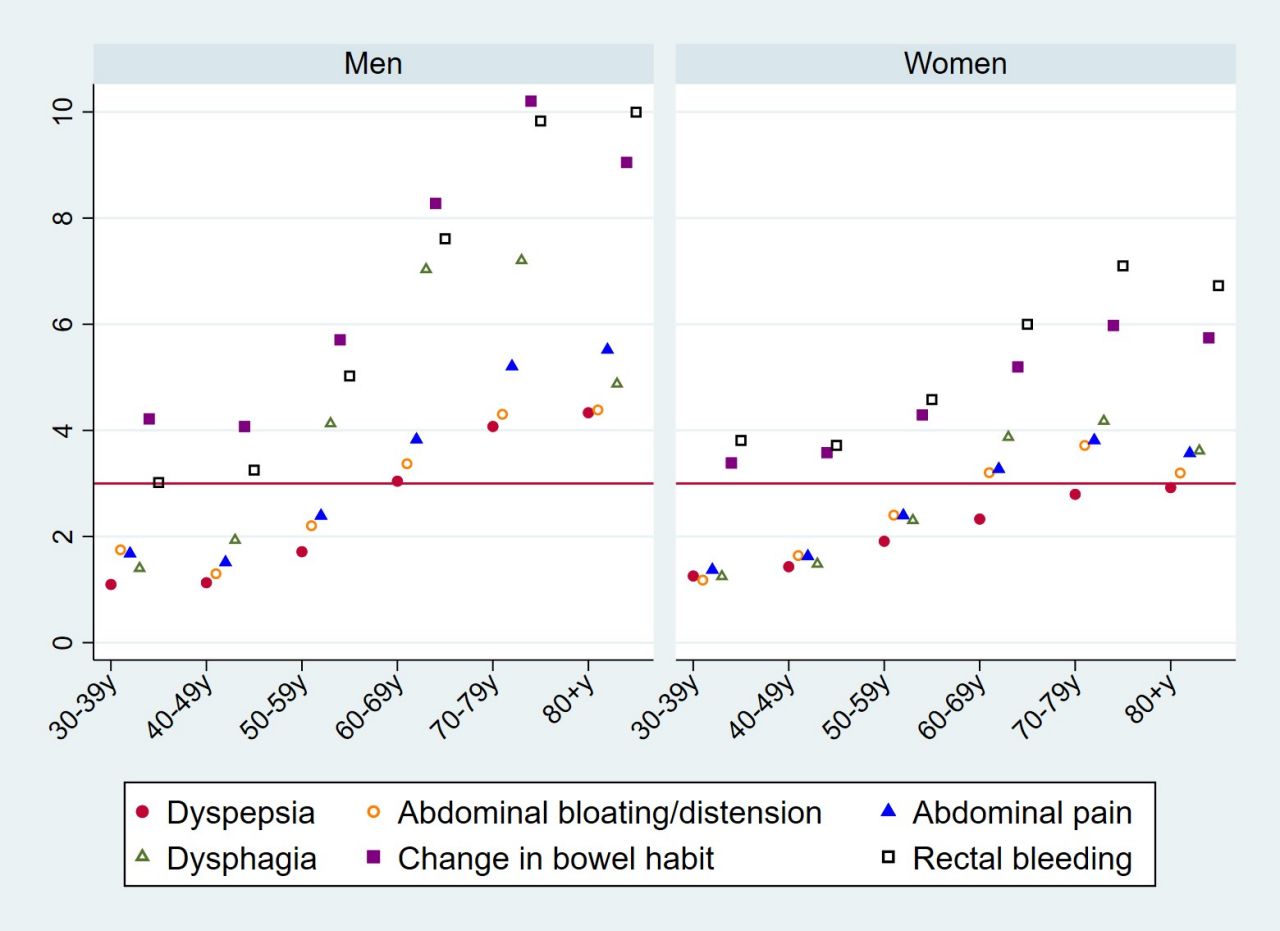
PPVs (%, *y* axis) for the composite of either cancer or IBD in the year post-presentation, per studied symptom, by sex (left/right panels) and age group (*x* axis). The red horizontal line represents the NICE 3% or greater PPV threshold for urgent (two-week-wait) referral for suspected cancer. Exact values are provided in S2 Table. IBD, inflammatory bowel disease; PPV, positive predictive value.

Considering the composite outcome of either cancer or IBD, the PPVs of dysphagia and rectal bleeding exceeded 3% across all age groups (**Fig 3**; see **S2 Table** for exact values), while those of abdominal pain, change in bowel habit, and dyspepsia exceeded 3% in patients aged 60 years or older.

Table 4 contextualises the number of observed cases of cancer and IBD compared with the number of cases that would have been expected by applying the 12-month general population incidence of these outcomes on the age and sex structure of the population of patients presenting with each symptom in our study.

**Table 4:** Illustration of additional cases of cancer and IBD following presentation with each studied symptom above what would be expected by the 12-month general population incidence of either condition, as applied to the age and sex composition of our study population.

	Cancer						IBD		
	Men			Women			Persons		
	Observed	Expected*	Additional	Observed	Expected*	Additional	Observed	Expected*	Additional
**Abdominal bloating/distension**	450	402	48	982	692	290	1,029	28.7	1,000
Per 1,000 patients**	16.5	14.7	1.8	13.3	9.4	3.9	10.2	0.3	9.9
**Abdominal pain**	5,841	4,282	1,559	6,749	5,082	1,667	10,386	257	10,129.3
Per 1,000 patients**	17.7	13.0	4.7	20.9	15.7	5.2	12	0.3	11.4
**Change in bowel habit**	2,101	879	1,222	1,471	838	633	2,859	29.5	2,829.5
Per 1,000 patients**	46.4	19.4	27.0	34.1	19.4	14.7	27	0.3	26.5
**Dyspepsia**	3,072	3,005	67	3,091	3,256	−165	4,939	146	4,792.5
Per 1,000 patients**	14.1	13.8	0.3	14.4	15.2	−0.8	10	0.3	9.3
**Dysphagia**	1,601	737	864	1,041	696	345	723	23.9	699.1
Per 1,000 patients**	42.8	19.7	23.1	29.1	19.4	9.6	8	0.3	8.1
**Rectal bleeding**	3,730	1,567	2,163	2,911	1,341	1,570	6,351	66.8	6,284.2
Per 1,000 patients**	32.0	13.4	18.5	25.8	11.9	13.9	27	0.3	26.7

As an example, 43 out of 1,000 men presenting with dysphagia would be diagnosed with cancer in the following year given the observed findings (see super-row 5 column 2, corresponding to PPV of 4.28% in Table 2). The corresponding number of cancer cases expected by applying the general age-specific population incidence would have been 20 (column 3). Therefore, dysphagia presentation is associated with 23 additional cases of cancer per 1,000 patients (column 4).

Across the symptom cohorts, it can be seen that the number of additional cancer cases is low for symptoms that are both associated with lower risk and younger median age at presentation (e.g., abdominal bloating/distension and dyspepsia, which have relatively lower PPVs for cancer (see Table 2) and lower median age at presentation in our study population of 53 years and 56, respectively; see Table 1). Conversely, the number of additional cases is greater for symptoms associated with relatively higher cancer risk and higher median age at presentation (e.g., change in bowel habit and dysphagia, which have relatively higher cancer risk; see Table 2 and median age at presentation of 63 years for both; see Table 1).

* Using age-specific population-based incidence estimates applied to the age (age/sex) structure of each of the 6 symptom cohorts.

** Per 1,000 patients presenting with the relevant symptom.

IBD, inflammatory bowel disease; PPV, positive predictive value.

**Table 5** displays PPVs for specific cancer sites, noting that the sum of PPVs across each row slightly exceeds the all-cancer PPVs reported in Table 2, as among the cohorts between 42 and 274 patients (0.02% to 0.16% of the cohorts or 1.9% to 4.8% of those diagnosed with any cancer) were diagnosed with more than 1 cancer type.

**Table 5:** Number of incident cases and PPVs (95% CIs) for cancer in the year following a studied symptom, by cancer site.

	**Any cancer**			**Colon**			**Rectal**			**Kidney**			**Stomach**		
**Men**	***n***	**PPV**	**(95% CI)**	***n***	**PPV**	**(95% CI)**	***n***	**PPV**	**(95% CI)**	***n***	**PPV**	**(95% CI)**	***n***	**PPV**	**(95% CI)**
Abdominal bloating/distension	450	1.65	(1.50, 1.80)	125	0.46	(0.38, 0.54)	25	0.09	(0.06, 0.13)	17	0.06	(0.03, 0.09)	24	0.09	(0.05, 0.12)
Abdominal pain	5,841	1.77	(1.73, 1.82)	1,733	0.53	(0.50, 0.55)	317	0.10	(0.09, 0.11)	217	0.07	(0.06, 0.07)	246	0.07	(0.07, 0.08)
Change in bowel habit	2,101	4.64	(4.45, 4.84)	815	1.80	(1.68, 1.92)	834	1.84	(1.72, 1.97)	35	0.08	(0.05, 0.10)	23	0.05	(0.03, 0.07)
Dyspepsia	3,072	1.41	(1.36, 1.46)	424	0.20	(0.18, 0.21)	122	0.06	(0.05, 0.07)	76	0.03	(0.03, 0.04)	298	0.14	(0.12, 0.15)
Dysphagia	1,601	4.28	(4.07, 4.48)	52	0.14	(0.10, 0.18)	17	0.05	(0.02, 0.07)	23	0.06	(0.04, 0.09)	133	0.36	(0.30, 0.42)
Rectal bleeding	3,730	3.20	(3.09, 3.30)	1,339	1.15	(1.09, 1.21)	1,453	1.24	(1.18, 1.31)	46	0.04	(0.03, 0.05)	48	0.04	(0.03, 0.05)
	**Any cancer**			**Colon**			**Rectal**			**Kidney**			**Stomach**		
**Women**	***n***	**PPV**	**(95% CI)**	***n***	**PPV**	**(95% CI)**	***n***	**PPV**	**(95% CI)**	***n***	**PPV**	**(95% CI)**	***n***	**PPV**	**(95% CI)**
Abdominal bloating/distension	982	1.33	(1.25, 1.42)	125	0.17	(0.14, 0.20)	24	0.03	(0.02, 0.05)	23	0.03	(0.02, 0.04)	12	0.02	(0.01, 0.03)
Abdominal pain	6,749	1.20	(1.17, 1.23)	1,651	0.29	(0.28, 0.31)	268	0.05	(0.04, 0.05)	200	0.04	(0.03, 0.04)	186	0.03	(0.03, 0.04)
Change in bowel habit	1,471	2.39	(2.27, 2.51)	561	0.91	(0.84, 0.99)	362	0.59	(0.53, 0.65)	19	0.03	(0.02, 0.04)	16	0.03	(0.01, 0.04)
Dyspepsia	3,091	1.03	(0.99, 1.07)	394	0.13	(0.12, 0.14)	97	0.03	(0.03, 0.04)	58	0.02	(0.01, 0.02)	153	0.05	(0.04, 0.06)
Dysphagia	1,041	2.13	(2.01, 2.26)	57	0.12	(0.09, 0.15)	19	0.04	(0.02, 0.06)	18	0.04	(0.02, 0.05)	53	0.11	(0.08, 0.14)
Rectal bleeding	2,911	2.46	(2.37, 2.55)	1,077	0.91	(0.86, 0.96)	1,088	0.92	(0.86, 0.97)	28	0.02	(0.01, 0.03)	27	0.02	(0.01, 0.03)
	**Lymphoma**			**Esophageal**			**Pancreatic**			**Sarcoma**			**Other** ^*^		
**Men**	***n***	**PPV**	**(95% CI)**	***n***	**PPV**	**(95% CI)**	***n***	**PPV**	**(95% CI)**	***n***	**PPV**	**(95% CI)**	***n***	**PPV**	**(95% CI)**
Abdominal bloating/distension	35	0.13	(0.09, 0.17)	15	0.05	(0.03, 0.08)	15	0.05	(0.03, 0.08)	<5	<0.02		211	0.77	(0.67, 0.88)
Abdominal pain	320	0.10	(0.09, 0.11)	317	0.10	(0.09, 0.11)	280	0.09	(0.08, 0.10)	9	0.00	(0.00, 0.00)	2,533	0.77	(0.74, 0.80)
Change in bowel habit	36	0.08	(0.05, 0.11)	23	0.05	(0.03, 0.07)	31	0.07	(0.04, 0.09)	<5	<0.01		411	0.91	(0.82, 1.00)
Dyspepsia	147	0.07	(0.06, 0.08)	556	0.26	(0.23, 0.28)	139	0.06	(0.05, 0.07)	8	0.00	(0.00, 0.01)	1,378	0.63	(0.60, 0.67)
Dysphagia	30	0.08	(0.05, 0.11)	1,024	2.74	(2.57, 2.90)	10	0.03	(0.01, 0.04)	5	0.01	(0.00, 0.03)	360	0.96	(0.86, 1.06)
Rectal bleeding	57	0.05	(0.04, 0.06)	64	0.05	(0.04, 0.07)	14	0.01	(0.01, 0.02)	<5	<0.004		859	0.74	(0.69, 0.78)
	**Lymphoma**			**Esophageal**			**Pancreatic**			**Sarcoma**			**Other** ^*^		
**Women**	***n***	**PPV**	**(95% CI)**	***n***	**PPV**	**(95% CI)**	***n***	**PPV**	**(95% CI)**	***n***	**PPV**	**(95% CI)**	***n***	**PPV**	**(95% CI)**
Abdominal bloating/distension	28	0.04	(0.02, 0.05)	10	0.01	(0.01, 0.02)	16	0.02	(0.01, 0.03)	<5	<0.01		337	0.46	(0.41, 0.51)
Abdominal pain	293	0.05	(0.05, 0.06)	162	0.03	(0.02, 0.03)	249	0.04	(0.04, 0.05)	16	0.00	(0.00, 0.00)	2,566	0.46	(0.44, 0.47)
Change in bowel habit	37	0.06	(0.04, 0.08)	10	0.02	(0.01, 0.03)	34	0.06	(0.04, 0.07)	<5	<0.01		368	0.60	(0.54, 0.66)
Dyspepsia	123	0.04	(0.03, 0.05)	242	0.08	(0.07, 0.09)	101	0.03	(0.03, 0.04)	10	0.00	(0.00, 0.01)	1,497	0.50	(0.47, 0.52)
Dysphagia	37	0.08	(0.05, 0.10)	516	1.06	(0.97, 1.15)	5	0.01	(0.00, 0.02)	<5	<0.01		327	0.67	(0.60, 0.74)
Rectal bleeding	45	0.04	(0.03, 0.05)	21	0.02	(0.01, 0.03)	21	0.02	(0.01, 0.03)	<5	<0.004		593	0.50	(0.46, 0.54)
					**Ovarian**					**Uterine**					
**Women**					***n***		**PPV**	**(95% CI)**		***n***		**PPV**		**(95% CI)**	
Abdominal bloating/distension					395		0.54	(0.48, 0.59)		34		0.05		(0.03, 0.06)	
Abdominal pain					1,043		0.19	(0.17, 0.20)		258		0.05		(0.04, 0.05)	
Change in bowel habit					100		0.16	(0.13, 0.19)		27		0.04		(0.03, 0.06)	
Dyspepsia					358		0.12	(0.11, 0.13)		102		0.03		(0.03, 0.04)	
Dysphagia					20		0.04	(0.02, 0.06)		12		0.02		(0.01, 0.04)	
Rectal bleeding					73		0.06	(0.05, 0.08)		60		0.05		(0.04, 0.06)	

* Comprising bladder, breast, cervical, laryngeal, thyroid, melanoma, myeloma, prostate, testicular, vulval, and vaginal.

CI, confidence interval; PPV, positive predictive value.

For change in bowel habit and rectal bleeding, the all-cancer PPVs in men (4.64% and 3.20%, respectively) were accounted for principally by colon (1.80% and 1.15%, respectively) and rectal cancers (1.84% and 1.24%, respectively), with similar patterns in women. Similarly, for dysphagia, the all-cancer PPV (4.28% in men and 2.13% in women) was principally accounted for by esophageal cancer (2.74% and 1.06%, respectively). For abdominal bloating/distension in women (all-cancer PPV: 1.33%), the PPV was highest for ovarian cancer (0.54%). Abdominal pain (all-cancer PPV of 1.77% in men and 1.20% in women) and dyspepsia (all-cancer PPV of 1.41% in men and 1.03% in women) had relatively low PPVs, which was accounted for by a broader range of cancer sites. The number of incident cases of cancer and PPVs for cancer (specific to colon, rectal, esophageal, and other cancers) stratified by age groups are provided in **S3 Table**.

Among cancer cases, the relative distribution of different cancer sites by symptom cohort (i.e., the cancer site case mix of each symptom cohort among diagnosed cases) is depicted in **S1 Fig**. Cancer cases diagnosed after presentation with either change in bowel habit or rectal bleeding were most likely diagnosed with colon and rectal cancer, whereas after presentation with dysphagia, esophageal was the most likely cancer diagnosis, noting, however, that across all 6 symptoms, a substantial minority of patients were diagnosed with “other” cancers. Considering patients diagnosed with either cancer or IBD, IBD contributed between 41% and 48% of all such cases for the 5 studied symptoms other than dysphagia, for which IBD was diagnosed in 21% (**S2 Fig**).

## Discussion

### Summary of findings

We estimated PPVs for cancer (overall, and of specific organs), for IBD, and for the composite outcome of either cancer or IBD, following a presentation to primary care with specific abdominal symptoms, by sex, age, and combination of the studied abdominal symptoms. PPVs for cancer were higher in men and older patients and in those presenting with change in bowel habit, dysphagia, or rectal bleeding. Cancer risk following change in bowel habit and rectal bleeding was mostly accounted for by colon and rectal cancer, while cancer risk following dysphagia was mostly accounted for by esophageal cancer; in women, cancer risk following abdominal distension/bloating was mostly accounted for by ovarian cancer. In men, cancer was more likely than IBD across all abdominal symptoms, while in women, IBD tended to be as or more likely than cancer. For the composite outcome of diagnosis of either cancer or IBD, PPVs of rectal bleeding exceeded the NICE-recommended specialist referral threshold of 3% in all ages and both sexes, as did PPVs of abdominal pain, change in bowel habit, and dyspepsia, in patients aged 60 years and over.

### Strengths and limitations

We estimated PPVs in 6 prospective symptom cohorts using data that are representative of the general population consulting in GP practice. We used comprehensive symptom code lists to capture abdominal symptoms, cancer, and IBD [9,14]. In defining the cohorts, we ensured that information was available for at least 1 year before and at least 1 year after the index consultation with the relevant symptom (to help identify “new” presentations and new diagnoses of cancer or IBD occurring post-presentation, as opposed to preexisting diagnoses of these conditions). Another strength of our study is the consideration of pairwise symptom combinations, describing how often they occur and how they moderate risk of cancer or IBD.

As common in all studies using primary care electronic health records, the identification of presenting symptoms and of the diagnoses of cancer or IBD relies on doctors recording them in patient records using appropriate codes. We only included data from patient records where the practice had met acceptable standards in THIN [16,17]. Our focus was to estimate PPVs in patients with abdominal symptoms that potentially exceed a threshold of concern for the GP, possibly triggering suspicion of cancer or other serious nonneoplastic disease such as IBD. Thus, our estimates represent PPVs among patients whose abdominal symptoms were deemed important to be coded in their records by their GPs. Future work should aim to incorporate additional features in risk stratification; these may include other presenting features and factors such as chronic morbidity and family history of cancer. Although cancer diagnoses could not be verified through linked cancer registration data, previous studies using similar primary care records have estimated that cancer diagnoses that remain unrecorded in primary care records are rare [16–18].

Our findings relate to general practice consultations within the National Health Service in the UK, characterised by a well-developed, free at point of access, primary healthcare system with a gatekeeping function, and where most patients experiencing symptoms present to primary care. Implicit consultation norms tend to favour “one principal complaint per consultation.” The findings may not be generalisable to other health systems where social norms about help seeking, and, therefore, risk levels associated with the same abdominal symptoms, may differ or where financial or structural barriers to accessing healthcare may exist. We included assessment of risk of IBD (in addition to assessment of risk of cancer, overall and by cancer site) given that it represents a consequential condition whose diagnostic pathway entails specialist assessment and endoscopic investigation, acknowledging that broader consideration of other nonneoplastic disease outcomes is also pertinent and should be addressed in future. When interpreting the findings, it is worth reflecting that they relate to patients with the studied symptoms who have presented to a GP. Consistent evidence from population studies in different countries indicates that larger proportions of the population experiencing such symptoms do not seek help [27–29]. Risk estimates among that broader population who experience the studied symptoms will be considerably lower than the ones reported among presenters. A small percentage of patients (1.2%) had a diagnosis of IBD recorded at the index consultation with one of the studied abdominal symptoms. One interpretation of this is that these patients were diagnosed with IBD in the past before the year preceding their consultation, and that the GP was rerecording their diagnosis; nonetheless, incidence patterns observed in our data concord with prior literature [25].

### Comparisons to other literature

With regard to PPVs for cancer, our findings overall concord with previous estimates [18,21]. However, unlike all but one previous study, we examine PPVs for the composite outcome of either cancer or IBD [8]. The single prior study of relevance estimated PPVs for a diagnosis of either colorectal cancer or IBD but was restricted to patients aged 50 years or younger and to 3 of the 6 abdominal symptoms considered in our study (abdominal pain, change in bowel habit, and rectal bleeding) [8]. Further, our study also includes both “all-cancer” and cancer site–specific evidence, whereas most prior literature in this field considered associations between specific abdominal symptoms and individual cancer sites in isolation. Our findings can be used in addition to other cancer risk tools in current use [30,31].

Our study adds to a small number of population-based studies examining the epidemiology of IBD. Concordant with prior literature, we observed a stable (although slightly declining) incidence in patients aged 30 years and over and an approximately equal risk among men and women [25]. Risk was greater (nearing 3%) among patients with rectal bleeding and change in bowel habit and lower although appreciable for patients with abdominal pain and abdominal bloating/distension (around 1%). Given known delays in the diagnosis of IBD [4], considering composite risk of either cancer or IBD diagnosis may help improve the timeliness of diagnosis of both diseases.

### Implications for policy, practice, and research

The findings of this comprehensive joint consideration of PPVs of 6 major abdominal symptoms for cancer (overall and site specific) and IBD have implications for clinical practice guidelines. Although cancers diagnosed following certain abdominal symptoms were mostly accounted for by a specific cancer site (e.g., colon and rectal cancer following change in bowel habit), among patients who were diagnosed with cancer after presentation with abdominal pain and dyspepsia, there was a diverse range of primary cancer sites involved. Understanding the relative risk of possible cancer sites (as denoted by the rank order of site-specific PPVs) can help guide the optimal testing strategies and support diagnostic decision-making regarding the use of primary care tests (such as fecal immunochemical testing) [21,22],or of referral to either rapid diagnostic centres (RDCs) or an appropriate clinical specialty. Further, it is worth considering the risk associated with specific symptoms across all cancer sites; in men, the 3% threshold for any cancer is exceeded for dysphagia in patients aged 50 years or older, for rectal bleeding and change in bowel habit in patients aged 60 years or older, and for the remaining 3 studied symptoms in patients 70 years or older. However, in women, the 3% risk for any cancer is exceeded only for dysphagia and rectal bleeding among women aged 60 years or older and for change in bowel habit among women 70 years or older.

Our findings could prove useful in considering risks of different cancer sites alongside the risk of other nonneoplastic disease. For example, in our study, PPVs of rectal bleeding for cancer and for IBD (in either sex) when considered in isolation were lower than 3% (i.e., lower than the threshold used in NICE clinical practice guidelines for recommending specialist fast track assessment). However, when PPVs were estimated for the composite outcome of either cancer or IBD, values exceeded the 3% threshold, across age groups, even in patients aged younger than 50 years. As the suspicion of abdominal cancer or of IBD often prompts similar diagnostic referral or investigation strategies, the findings would support reconsideration of current guideline recommendations, enabling specialist referrals even when cancer-specific PPVs of presenting abdominal symptoms do not surpass the normative 3% threshold when considered in isolation.

## Supporting information

S1 Text STROBE Statement. STROBE, STrengthening the Reporting of OBservational studies in Epidemiology.
(DOCX)

S2 Text Analysis plan. (DOCX)

S1 Table Numbers of incident cases and PPVs (%) for cancer and for IBD within 1 year of symptom, per type of symptom, by sex and age group. IBD, inflammatory bowel disease; PPV, positive predictive value.
(DOCX)

S2 Table Numbers of incident cases and PPVs (%) for either cancer or IBD within 1 year of symptom, per type of symptom, by sex and age group. IBD, inflammatory bowel disease; PPV, positive predictive value.
(DOCX)

S3 Table Numbers of incident cases and PPVs (%) for cancer, by cancer site (specific to colon, rectal, esophageal and other cancers, stratified by age group), within 1 year of symptom, per type of symptom, stratified by age group. PPV, positive predictive value.
(DOCX)

S1 Fig The relative distribution of different cancer sites (among cases diagnosed with cancer) for each abdominal symptom cohort. (DOCX)

S2 Fig The relative distribution of any cancer diagnosis or IBD among for each abdominal symptom cohort. IBD, inflammatory bowel disease.
(DOCX)

S1 Diagram Derivation of each of the 6 symptom cohorts. * No records during 2000–2016 where the patient was 30–99 years old, where patient has been registered with the practice for at least 6 months and practice has achieved current recording quality standards, and 1 year before any transfer or date when the practice last provided data to THIN. THIN, The Health Improvement Network.
(PDF)

## Abbreviations

CI: confidence interval
GP: general practitioner
IBD: inflammatory bowel disease
NICE: National Institute of Health and Care Excellence
PPV: positive predictive value
RDC: rapid diagnostic centre
STROBE: STrengthening the Reporting of OBservational studies in Epidemiology
THIN: The Health Improvement Network
