# Supplementary material for: Predictive values for different cancers and inflammatory bowel disease of 6 common abdominal symptoms among more than 1.9 million primary care patients in the UK: A cohort study
Source: PLoS Med. 2021 Aug 2;18(8):e1003708. doi: 10.1371/journal.pmed.1003708 (PMC8367005; doi:10.1371/journal.pmed.1003708)
Supplement: S1 Text — STROBE, STrengthening the Reporting of OBservational studies in Epidemiology. (DOCX) [file pmed.1003708.s001.docx]

STROBE Statement—checklist of items that should be included in reports of observational studies

|  | Item No | Recommendation | Page  No |
| --- | --- | --- | --- |
| **Title and abstract** | 1 | (*a*) Indicate the study’s design with a commonly used term in the title or the abstract | See Title. |
|  |  | (*b*) Provide in the abstract an informative and balanced summary of what was done and what was found | See Abstract. |
| Introduction | | | |
| Background/rationale | 2 | Explain the scientific background and rationale for the investigation being reported | See paras 1-2 of Introduction. |
| Objectives | 3 | State specific objectives, including any prespecified hypotheses | See para 3 of Introduction. |
| Methods | | | |
| Study design | 4 | Present key elements of study design early in the paper | See section entitled ‘Study design’ within Methods, together with Figure 1 (cohort derivation). |
| Setting | 5 | Describe the setting, locations, and relevant dates, including periods of recruitment, exposure, follow-up, and data collection | See section entitled ‘Study design’ within Methods (paras 1-4). |
| Participants | 6 | (*a*) *Cohort study*—Give the eligibility criteria, and the sources and methods of selection of participants. Describe methods of follow-up  *Case-control study*—Give the eligibility criteria, and the sources and methods of case ascertainment and control selection. Give the rationale for the choice of cases and controls  *Cross-sectional study*—Give the eligibility criteria, and the sources and methods of selection of participants | See section entitled ‘Study design’ within Methods (paras 1-4). |
|  |  | (*b*) *Cohort study*—For matched studies, give matching criteria and number of exposed and unexposed  *Case-control study*—For matched studies, give matching criteria and the number of controls per case | Not applicable. |
| Variables | 7 | Clearly define all outcomes, exposures, predictors, potential confounders, and effect modifiers. Give diagnostic criteria, if applicable | Please see paragraph entitled ‘Exposure, outcomes and other covariates’ within Methods. |
| Data sources/ measurement | 8* | For each variable of interest, give sources of data and details of methods of assessment (measurement). Describe comparability of assessment methods if there is more than one group | See section entitled ‘Study design’ within Methods (paras 1-4) |
| Bias | 9 | Describe any efforts to address potential sources of bias | See section entitled ‘Study design’ within Methods, paragraphs 2-4. |
| Study size | 10 | Explain how the study size was arrived at | Please see ‘Study design’ within Methods, paragraph 1. |
| Quantitative variables | 11 | Explain how quantitative variables were handled in the analyses. If applicable, describe which groupings were chosen and why | Not applicable in our study as principal exposures (symptoms) and outcomes (diagnosis of cancer or inflammatory bowel disease) were discrete. |
| Statistical methods | 12 | (*a*) Describe all statistical methods, including those used to control for confounding | Please see ‘Statistical analysis’ within Methods. |
|  |  | (*b*) Describe any methods used to examine subgroups and interactions | We have stratified by sex and age a priori, as prior evidence indicates large variation in positive predictive values by demographic strata and clinical guidelines are also specified by age group and sex. |
|  |  | (*c*) Explain how missing data were addressed | Not applicable in our study as principal exposures (symptoms) and outcomes (disease occurrence) were assumed complete. No statistical treatment would have been applicable in our study, but the issue is acknowledged in limitations section. |
|  |  | (*d*) *Cohort study*—If applicable, explain how loss to follow-up was addressed  *Case-control study*—If applicable, explain how matching of cases and controls was addressed  *Cross-sectional study*—If applicable, describe analytical methods taking account of sampling strategy | Not applicable. |
|  |  | (*e*) Describe any sensitivity analyses | Not applicable. |

Continued on next page

| Results | | | |
| --- | --- | --- | --- |
| Participants | 13* | (a) Report numbers of individuals at each stage of study—eg numbers potentially eligible, examined for eligibility, confirmed eligible, included in the study, completing follow-up, and analysed | See Figure 1. |
|  |  | (b) Give reasons for non-participation at each stage | See Figure 1 and ‘Study design’ section within Methods. |
|  |  | (c) Consider use of a flow diagram | See Figure 1 which is a flow diagram. |
| Descriptive data | 14* | (a) Give characteristics of study participants (eg demographic, clinical, social) and information on exposures and potential confounders | Please see ‘Study population and patient characteristics’ within ‘Results’, and also Table 1. |
|  |  | (b) Indicate number of participants with missing data for each variable of interest | Please see ‘Study population and patient characteristics’ within ‘Results’, and also Table 1. |
|  |  | (c) *Cohort study*—Summarise follow-up time (eg, average and total amount) | All participants were followed-up for 12 months as stipulated in Methods, ‘Study design’. |
| Outcome data | 15* | *Cohort study*—Report numbers of outcome events or summary measures over time | Reported in Tables 2, 3 and 5 and Figures 2-4 and related supplementary files and sections of Results (main text). |
|  |  | *Case-control study—*Report numbers in each exposure category, or summary measures of exposure | Not applicable. |
|  |  | *Cross-sectional study—*Report numbers of outcome events or summary measures | Not applicable. |
| Main results | 16 | (*a*) Give unadjusted estimates and, if applicable, confounder-adjusted estimates and their precision (eg, 95% confidence interval). Make clear which confounders were adjusted for and why they were included | Reported in Tables 2, 3 and 5 and Figures 2-4 and related supplementary files and sections of Results (main text). |
|  |  | (*b*) Report category boundaries when continuous variables were categorized | Not applicable. |
|  |  | (*c*) If relevant, consider translating estimates of relative risk into absolute risk for a meaningful time period | Not applicable. |
| Other analyses | 17 | Report other analyses done—eg analyses of subgroups and interactions, and sensitivity analyses | Not applicable. |
| Discussion | | | |
| Key results | 18 | Summarise key results with reference to study objectives | See ‘Summary of findings’ in Discussion. |
| Limitations | 19 | Discuss limitations of the study, taking into account sources of potential bias or imprecision. Discuss both direction and magnitude of any potential bias | See ‘Strengths and limitations’ in Discussion. |
| Interpretation | 20 | Give a cautious overall interpretation of results considering objectives, limitations, multiplicity of analyses, results from similar studies, and other relevant evidence | See ‘Implications for policy, practice and research’ in Discussion. |
| Generalisability | 21 | Discuss the generalisability (external validity) of the study results | See ‘Implications….’ And ‘Strengths and limitations’ sections in Discussion. |
| Other information | | | |
| Funding | 22 | Give the source of funding and the role of the funders for the present study and, if applicable, for the original study on which the present article is based | Provided in article meta-data. |

*Give information separately for cases and controls in case-control studies and, if applicable, for exposed and unexposed groups in cohort and cross-sectional studies.

**Note:** An Explanation and Elaboration article discusses each checklist item and gives methodological background and published examples of transparent reporting. The STROBE checklist is best used in conjunction with this article (freely available on the Web sites of PLoS Medicine at http://www.plosmedicine.org/, Annals of Internal Medicine at http://www.annals.org/, and Epidemiology at http://www.epidem.com/). Information on the STROBE Initiative is available at www.strobe-statement.org.
