# Supplementary material for: Predictive values for different cancers and inflammatory bowel disease of 6 common abdominal symptoms among more than 1.9 million primary care patients in the UK: A cohort study
Source: PLoS Med. 2021 Aug 2;18(8):e1003708. doi: 10.1371/journal.pmed.1003708 (PMC8367005; doi:10.1371/journal.pmed.1003708)
Supplement: S2 Text — (DOCX) [file pmed.1003708.s002.docx]

**Understanding the predictive values of common abdominal symptoms for cancer and inflammatory bowel disease in primary care consultees: Analysis plan**

This analysis plan has been written in the order that it is expected to be carried out. For example, the dataset should be cleaned (C.) before the cohort subset is derived from the inclusion/exclusion criteria (D.)

1. **Research question(s) and/or hypotheses**

Our main research questions are:

1. What is the risk of cancer within the next year, given that a patient presents to the GP with abdominal symptom X? Where X is one of 6 different symptoms.
2. What is the risk of inflammatory bowel disease with the next year, given that a patient presents to the GP with abdominal symptom X?
3. What is the risk of a cancer/inflammatory bowel disease with the next year, given that a patient presents to the GP with a pair-wise combination of the studied abdominal symptoms?
4. **Datasets to be used**

The Health Improvement Network (THIN) data for (1^st^ Jan 2000 – 31^st^ Dec 2015).

1. **Data preparation / specification / cleaning**

- Data on symptoms at presentation are extracted by using relevant Read code lists and searching the medical records for codes indicating the conditions of interest.
- For covariates: data for sex and year of birth (to derive age at presentation) will be extracted from the patient records and checked for missing values.

For each patient, the follow-up period will be defined as end date – start date, where:

- *Start date*, the latest of: date of registration with the practice, acceptable computer usage (ACU) and acceptable mortality rate (AMR) dates, first date of study period, year of birth + 30*365.25]; and
- *End date*, the earliest of: date of transfer out, date of last data recording from the practice, date of death, last date of study period, year of birth + 99*365.25]

1. **Inclusion/exclusion criteria**

Inclusion criteria: Patients who present with one of 6 abdominal symptoms the study period

Exclusion criteria: Patients aged less than 30 years old; patients who have presented with any of the studied 6 symptoms in the previous year; Patients with missing year of birth, sex, those who are not permanently registered with the practice (variable patflag does not take values A or C), and those with end date < start date (see below) will be excluded.

1. **Variables to be used in the main analysis**

Exposure variable: each of the 6 abdominal symptoms symptoms (in turn)

Outcome variable: diagnosis of cancer or inflammatory bowel disease in the year following the presenting symptom.

Covariates: sex, age, deprivation, ethnicity, body mass index, tobacco use, alcohol intake, comorbidities, prescriptions, investigations.

1. **Establishing codes/groupings for all exposures, outcomes, and covariates**

Codes for the 6 symptoms of interest (the exposures), and diagnosis of cancer and inflammatory bowel disease (the outcomes) will be used as previously developed by co-authors WH and SP.

Results will be presented both overall and by cancer site, where cancer sites are the most common cancers among men, and among women, i.e.: prostate, lung, bowel, head and neck, melanoma, kidney, Non-Hodgkin’s Lymphoma, bladder, leukaemia, oesophageal, breast, uterine, ovarian, pancreatic. Other cancer sites will be grouped as ‘other’.

1. **Statistical methods and software to be used**
2. *What is the risk of cancer within the next year, given that a patient presents to the GP with abdominal symptom X?*

First PPVs will be calculated as the number of patients who have a given symptom and are then diagnosed with cancer in the following year, divided by the number of patients who have the presented with the given symptom. Confidence intervals will be calculated. PPVs will also, as a minimum, be estimated by sex-age subgroups.

1. *What is the risk of inflammatory bowel disease within the next year, given that a patient presents to the GP with abdominal symptom X?*

PPVs and their confidence intervals will be calculated as in 1. (where outcome was cancer), but this time where outcome is inflammatory bowel disease.

1. *What is the risk of a cancer/inflammatory bowel disease within the next year, given that a patient presents to the GP with more than one of the studied symptoms?*

We will examine such associations for pair-wise combinations of the 6 studied symptoms.

We will be analysing the data using Stata.

1. **Draft Results tables**

Table 1. Characteristics of patients in cohort at time of presenting symptom

| **Demographic variables** | **n (%) or median (IQR)** |
| --- | --- |
| Sex |  |
| Female |  |
| Age at time of index presentation |  |
| 30-39y |  |
| 40-49y |  |
| 50-59y |  |
| 60-69y |  |
| 70-79y |  |
| 80+y |  |
|  |  |
| **Clinical variables** |  |
| Symptom(s)* |  |
| Abdominal pain |  |
| Abdominal bloating |  |
| Change in bowel habit |  |
| Dyspepsia |  |
| Dysphagia |  |
| Rectal bleeding |  |

*Note patients can have multiple symptoms at their index presentation and therefore %s will not add to 100.

**NB: Tables 2 and 3 are provided for initial conceptual mapping.**

**Table 2. Positive predictive values (95% Confidence Intervals) of cancer or inflammatory bowel disease in the year following a symptom**

|  | **Men** | | | | **Women** | | | |
| --- | --- | --- | --- | --- | --- | --- | --- | --- |
| **Symptom** | **Cancer** | | **Inflammatory bowel disease** | | **Cancer** | | **Inflammatory bowel disease** | |
| Abdominal pain |  |  |  |  |  |  |  |  |
| Abdominal bloating |  |  |  |  |  |  |  |  |
| Breast lump |  |  |  |  |  |  |  |  |
| Change in bowel habit |  |  |  |  |  |  |  |  |
| Dyspepsia |  |  |  |  |  |  |  |  |
| Rectal bleeding |  |  |  |  |  |  |  |  |

**Figure: Positive predictive values plotted for cancer/inflammatory bowel disease by sex and age group**

**Table 3. Positive predictive values (95% Confidence Intervals) of cancer in the year following a symptom, by cancer site**

| **Symptoms in men** | **Prostate** | | **Lung** | | **Bowel** | | **Head and neck** | | **Melanoma** | | **Kidney** | | **Non-Hodgkin’s Lymphoma** | | **Bladder** | | **Leukaemia** | | **Oesophageal** | | **Other** | |
| --- | --- | --- | --- | --- | --- | --- | --- | --- | --- | --- | --- | --- | --- | --- | --- | --- | --- | --- | --- | --- | --- | --- |
| Abdominal pain |  |  |  |  |  |  |  |  |  |  |  |  |  |  |  |  |  |  |  |  |  |  |
| Abdominal bloating |  |  |  |  |  |  |  |  |  |  |  |  |  |  |  |  |  |  |  |  |  |  |
| Change in bowel habit |  |  |  |  |  |  |  |  |  |  |  |  |  |  |  |  |  |  |  |  |  |  |
| Dyspepsia |  |  |  |  |  |  |  |  |  |  |  |  |  |  |  |  |  |  |  |  |  |  |
| Dysphagia |  |  |  |  |  |  |  |  |  |  |  |  |  |  |  |  |  |  |  |  |  |  |
| Rectal bleeding |  |  |  |  |  |  |  |  |  |  |  |  |  |  |  |  |  |  |  |  |  |  |
| **Symptoms in women** | **Breast** | | **Lung** | | **Bowel** | | **Uterine** | | **Melanoma** | | **Kidney** | | **Non-Hodgkin’s Lymphoma** | | **Ovarian** | | **Brain/ CNS/ Intracranial endocrine** | | **Pancreatic** | | **Other** | |
| Abdominal pain |  |  |  |  |  |  |  |  |  |  |  |  |  |  |  |  |  |  |  |  |  |  |
| Abdominal bloating |  |  |  |  |  |  |  |  |  |  |  |  |  |  |  |  |  |  |  |  |  |  |
| Breast lump |  |  |  |  |  |  |  |  |  |  |  |  |  |  |  |  |  |  |  |  |  |  |
| Change in bowel habit |  |  |  |  |  |  |  |  |  |  |  |  |  |  |  |  |  |  |  |  |  |  |
| Dyspepsia |  |  |  |  |  |  |  |  |  |  |  |  |  |  |  |  |  |  |  |  |  |  |
| Rectal bleeding |  |  |  |  |  |  |  |  |  |  |  |  |  |  |  |  |  |  |  |  |  |  |
