# Supplementary material for: Predictive values for different cancers and inflammatory bowel disease of 6 common abdominal symptoms among more than 1.9 million primary care patients in the UK: A cohort study
Source: PLoS Med. 2021 Aug 2;18(8):e1003708. doi: 10.1371/journal.pmed.1003708 (PMC8367005; doi:10.1371/journal.pmed.1003708)
Supplement: S1 Table — IBD, inflammatory bowel disease; PPV, positive predictive value. (DOCX) [file pmed.1003708.s003.docx]

**Supplementary Table S1. Numbers of incident cases and positive predictive values (%) for cancer and for IBD within one year of symptom, per type of symptom, by sex and age group**

| **Outcome: sex**  Symptom | **n** | **PPV** | **(95% CI)** | **n** | **PPV** | **(95% CI)** | **n** | **PPV** | **(95% CI)** |
| --- | --- | --- | --- | --- | --- | --- | --- | --- | --- |
| **Cancer: Men** | **Age 30-39** |  |  | **Age 40-49** |  |  | **Age 50-59** |  |  |
| Abdominal bloating/ distension | 7 | 0.20 | (0.05, 0.34) | 10 | 0.18 | (0.07, 0.30) | 67 | 1.13 | (0.86, 1.40) |
| Abdominal pain | 87 | 0.14 | (0.11, 0.17) | 303 | 0.42 | (0.38, 0.47) | 894 | 1.30 | (1.22, 1.39) |
| Change in bowel habit | 13 | 0.45 | (0.20, 0.69) | 60 | 0.98 | (0.73, 1.22) | 266 | 2.74 | (2.42, 3.06) |
| Dyspepsia | 35 | 0.10 | (0.07, 0.14) | 117 | 0.26 | (0.21, 0.30) | 407 | 0.89 | (0.80, 0.97) |
| Dysphagia | 8 | 0.26 | (0.08, 0.44) | 65 | 1.18 | (0.90, 1.47) | 237 | 3.28 | (2.87, 3.70) |
| Rectal bleeding | 34 | 0.17 | (0.11, 0.23) | 179 | 0.70 | (0.60, 0.81) | 614 | 2.46 | (2.27, 2.66) |
|  | **Age 60-69** |  |  | **Age 70-79** |  |  | **Age 80+** |  |  |
| Abdominal bloating/ distension | 153 | 2.49 | (2.10, 2.88) | 148 | 3.41 | (2.87, 3.95) | 65 | 3.52 | (2.68, 4.36) |
| Abdominal pain | 1769 | 2.80 | (2.67, 2.93) | 1896 | 4.23 | (4.04, 4.42) | 892 | 4.44 | (4.15, 4.72) |
| Change in bowel habit | 604 | 5.40 | (4.98, 5.82) | 780 | 7.70 | (7.18, 8.22) | 378 | 7.32 | (6.61, 8.03) |
| Dyspepsia | 1007 | 2.15 | (2.02, 2.29) | 1036 | 3.20 | (3.01, 3.39) | 470 | 3.56 | (3.24, 3.88) |
| Dysphagia | 556 | 6.38 | (5.87, 6.89) | 511 | 6.52 | (5.97, 7.06) | 224 | 4.39 | (3.83, 4.95) |
| Rectal bleeding | 1077 | 4.80 | (4.52, 5.08) | 1200 | 7.29 | (6.89, 7.69) | 626 | 8.18 | (7.57, 8.79) |
| **Cancer: Women** | **Age 30-39** |  |  | **Age 40-49** |  |  | **Age 50-59** |  |  |
| Abdominal bloating/ distension | 23 | 0.16 | (0.10, 0.23) | 115 | 0.62 | (0.51, 0.74) | 216 | 1.42 | (1.23, 1.61) |
| Abdominal pain | 244 | 0.18 | (0.15, 0.20) | 705 | 0.56 | (0.52, 0.60) | 1357 | 1.29 | (1.22, 1.36) |
| Change in bowel habit | 13 | 0.35 | (0.16, 0.53) | 83 | 0.98 | (0.77, 1.19) | 255 | 1.90 | (1.67, 2.14) |
| Dyspepsia | 74 | 0.16 | (0.12, 0.20) | 278 | 0.46 | (0.41, 0.52) | 587 | 0.94 | (0.87, 1.02) |
| Dysphagia | 10 | 0.25 | (0.10, 0.41) | 52 | 0.72 | (0.53, 0.92) | 134 | 1.44 | (1.20, 1.69) |
| Rectal bleeding | 54 | 0.28 | (0.21, 0.36) | 190 | 0.86 | (0.73, 0.98) | 527 | 2.20 | (2.01, 2.38) |
|  | **Age 60-69** |  |  | **Age 70-79** |  |  | **Age 80+** |  |  |
| Abdominal bloating/ distension | 278 | 2.34 | (2.07, 2.61) | 244 | 2.71 | (2.37, 3.04) | 106 | 2.17 | (1.76, 2.58) |
| Abdominal pain | 1930 | 2.17 | (2.08, 2.27) | 1656 | 2.55 | (2.43, 2.67) | 857 | 2.30 | (2.15, 2.46) |
| Change in bowel habit | 387 | 2.58 | (2.33, 2.84) | 471 | 3.52 | (3.21, 3.84) | 262 | 3.48 | (3.06, 3.89) |
| Dyspepsia | 861 | 1.40 | (1.31, 1.50) | 846 | 1.82 | (1.70, 1.95) | 445 | 1.82 | (1.65, 1.99) |
| Dysphagia | 297 | 3.08 | (2.73, 3.42) | 317 | 3.26 | (2.91, 3.61) | 231 | 2.58 | (2.25, 2.91) |
| Rectal bleeding | 732 | 3.36 | (3.12, 3.60) | 824 | 4.57 | (4.27, 4.88) | 584 | 4.44 | (4.09, 4.80) |
| **IBD: Men** | **Age 30-39** |  |  | **Age 40-49** |  |  | **Age 50-59** |  |  |
| Abdominal bloating/ distension | 56 | 1.58 | (1.17, 1.99) | 61 | 1.12 | (0.84, 1.40) | 65 | 1.09 | (0.83, 1.36) |
| Abdominal pain | 932 | 1.54 | (1.44, 1.64) | 786 | 1.10 | (1.02, 1.17) | 763 | 1.11 | (1.03, 1.19) |
| Change in bowel habit | 111 | 3.81 | (3.11, 4.50) | 192 | 3.13 | (2.69, 3.56) | 290 | 2.99 | (2.65, 3.33) |
| Dyspepsia | 335 | 1.00 | (0.89, 1.11) | 398 | 0.87 | (0.79, 0.96) | 386 | 0.84 | (0.76, 0.92) |
| Dysphagia | 35 | 1.14 | (0.77, 1.52) | 41 | 0.75 | (0.52, 0.98) | 64 | 0.89 | (0.67, 1.10) |
| Rectal bleeding | 564 | 2.85 | (2.62, 3.08) | 650 | 2.55 | (2.36, 2.75) | 644 | 2.58 | (2.39, 2.78) |
|  | **Age 60-69** |  |  | **Age 70-79** |  |  | **Age 80+** |  |  |
| Abdominal bloating/ distension | 55 | 0.90 | (0.66, 1.13) | 43 | 0.99 | (0.70, 1.28) | 16 | 0.87 | (0.44, 1.29) |
| Abdominal pain | 668 | 1.06 | (0.98, 1.14) | 468 | 1.04 | (0.95, 1.14) | 225 | 1.12 | (0.97, 1.27) |
| Change in bowel habit | 330 | 2.95 | (2.64, 3.26) | 260 | 2.57 | (2.26, 2.88) | 95 | 1.84 | (1.47, 2.21) |
| Dyspepsia | 422 | 0.90 | (0.82, 0.99) | 295 | 0.91 | (0.81, 1.02) | 105 | 0.80 | (0.64, 0.95) |
| Dysphagia | 59 | 0.68 | (0.50, 0.85) | 56 | 0.71 | (0.53, 0.90) | 27 | 0.53 | (0.33, 0.73) |
| Rectal bleeding | 641 | 2.86 | (2.64, 3.08) | 433 | 2.63 | (2.39, 2.88) | 148 | 1.93 | (1.63, 2.24) |
| **IBD: Women** | **Age 30-39** |  |  | **Age 40-49** |  |  | **Age 50-59** |  |  |
| Abdominal bloating/ distension | 144 | 1.02 | (0.85, 1.18) | 189 | 1.02 | (0.88, 1.17) | 150 | 0.99 | (0.83, 1.15) |
| Abdominal pain | 1665 | 1.20 | (1.15, 1.26) | 1369 | 1.08 | (1.02, 1.14) | 1174 | 1.12 | (1.05, 1.18) |
| Change in bowel habit | 116 | 3.09 | (2.54, 3.65) | 222 | 2.63 | (2.28, 2.97) | 326 | 2.43 | (2.17, 2.69) |
| Dyspepsia | 505 | 1.10 | (1.01, 1.20) | 582 | 0.97 | (0.89, 1.05) | 602 | 0.97 | (0.89, 1.05) |
| Dysphagia | 39 | 0.99 | (0.68, 1.30) | 55 | 0.76 | (0.56, 0.96) | 82 | 0.88 | (0.69, 1.07) |
| Rectal bleeding | 682 | 3.55 | (3.28, 3.81) | 637 | 2.87 | (2.65, 3.09) | 586 | 2.45 | (2.25, 2.64) |
|  | **Age 60-69** |  |  | **Age 70-79** |  |  | **Age 80+** |  |  |
| Abdominal bloating/ distension | 108 | 0.91 | (0.74, 1.08) | 91 | 1.01 | (0.80, 1.22) | 51 | 1.05 | (0.76, 1.33) |
| Abdominal pain | 1007 | 1.13 | (1.06, 1.20) | 849 | 1.31 | (1.22, 1.39) | 480 | 1.29 | (1.18, 1.40) |
| Change in bowel habit | 399 | 2.66 | (2.41, 2.92) | 341 | 2.55 | (2.28, 2.82) | 177 | 2.35 | (2.01, 2.69) |
| Dyspepsia | 574 | 0.94 | (0.86, 1.01) | 459 | 0.99 | (0.90, 1.08) | 276 | 1.13 | (1.00, 1.26) |
| Dysphagia | 77 | 0.80 | (0.62, 0.97) | 93 | 0.96 | (0.76, 1.15) | 95 | 1.06 | (0.85, 1.27) |
| Rectal bleeding | 592 | 2.72 | (2.50, 2.93) | 464 | 2.58 | (2.34, 2.81) | 310 | 2.36 | (2.10, 2.62) |
