# Supplementary material for: Predictive values for different cancers and inflammatory bowel disease of 6 common abdominal symptoms among more than 1.9 million primary care patients in the UK: A cohort study
Source: PLoS Med. 2021 Aug 2;18(8):e1003708. doi: 10.1371/journal.pmed.1003708 (PMC8367005; doi:10.1371/journal.pmed.1003708)
Supplement: S2 Table — IBD, inflammatory bowel disease; PPV, positive predictive value. (DOCX) [file pmed.1003708.s004.docx]

**Supplementary Table S2. Numbers of incident cases and positive predictive values (%) for either cancer or IBD within one year of symptom, per type of symptom, by sex and age group**

| **Sex**  Symptom | **n** | **PPV** | **(95% CI)** | **n** | **PPV** | **(95% CI)** | **n** | **PPV** | **(95% CI)** |
| --- | --- | --- | --- | --- | --- | --- | --- | --- | --- |
| **Men** | **Age 30-39** |  |  | **Age 40-49** |  |  | **Age 50-59** |  |  |
| Abdominal bloating/ distension | 62 | 1.75 | (1.32, 2.18) | 71 | 1.30 | (1.00, 1.60) | 131 | 2.21 | (1.83, 2.58) |
| Abdominal pain | 1017 | 1.68 | (1.58, 1.78) | 1086 | 1.51 | (1.43, 1.60) | 1644 | 2.39 | (2.28, 2.51) |
| Change in bowel habit | 123 | 4.22 | (3.49, 4.95) | 250 | 4.07 | (3.58, 4.57) | 554 | 5.71 | (5.24, 6.17) |
| Dyspepsia | 368 | 1.10 | (0.99, 1.21) | 515 | 1.13 | (1.03, 1.23) | 787 | 1.71 | (1.60, 1.83) |
| Dysphagia | 43 | 1.40 | (0.99, 1.82) | 106 | 1.93 | (1.57, 2.30) | 298 | 4.13 | (3.67, 4.59) |
| Rectal bleeding | 598 | 3.02 | (2.78, 3.26) | 828 | 3.25 | (3.03, 3.47) | 1252 | 5.03 | (4.75, 5.30) |
|  | **Age 60-69** |  |  | **Age 70-79** |  |  | **Age 80+** |  |  |
| Abdominal bloating/ distension | 207 | 3.37 | (2.92, 3.82) | 187 | 4.30 | (3.70, 4.91) | 81 | 4.39 | (3.45, 5.32) |
| Abdominal pain | 2418 | 3.83 | (3.68, 3.98) | 2333 | 5.21 | (5.00, 5.41) | 1109 | 5.52 | (5.20, 5.83) |
| Change in bowel habit | 926 | 8.28 | (7.77, 8.79) | 1033 | 10.20 | (9.61, 10.79) | 467 | 9.05 | (8.27, 9.83) |
| Dyspepsia | 1423 | 3.04 | (2.89, 3.20) | 1318 | 4.07 | (3.86, 4.29) | 572 | 4.33 | (3.99, 4.68) |
| Dysphagia | 613 | 7.03 | (6.50, 7.57) | 565 | 7.20 | (6.63, 7.78) | 249 | 4.88 | (4.29, 5.47) |
| Rectal bleeding | 1707 | 7.61 | (7.26, 7.96) | 1618 | 9.83 | (9.38, 10.29) | 765 | 10.00 | (9.32, 10.67) |
| **Women** | **Age 30-39** |  |  | **Age 40-49** |  |  | **Age 50-59** |  |  |
| Abdominal bloating/ distension | 167 | 1.178713 | (1.00, 1.36) | 303 | 1.64 | (1.46, 1.83) | 365 | 2.40 | (2.16, 2.65) |
| Abdominal pain | 1900 | 1.373477 | (1.31, 1.43) | 2063 | 1.63 | (1.56, 1.70) | 2519 | 2.40 | (2.30, 2.49) |
| Change in bowel habit | 127 | 3.386667 | (2.81, 3.97) | 303 | 3.58 | (3.19, 3.98) | 575 | 4.29 | (3.95, 4.64) |
| Dyspepsia | 577 | 1.257355 | (1.16, 1.36) | 857 | 1.43 | (1.34, 1.53) | 1188 | 1.91 | (1.80, 2.02) |
| Dysphagia | 49 | 1.249362 | (0.90, 1.60) | 107 | 1.48 | (1.20, 1.76) | 214 | 2.31 | (2.00, 2.61) |
| Rectal bleeding | 733 | 3.811356 | (3.54, 4.08) | 826 | 3.72 | (3.47, 3.97) | 1098 | 4.58 | (4.32, 4.85) |
| **Women** | **Age 60-69** |  |  | **Age 70-79** |  |  | **Age 80+** |  |  |
| Abdominal bloating/ distension | 381 | 3.20 | (2.89, 3.52) | 335 | 3.72 | (3.33, 4.11) | 156 | 3.20 | (2.70, 3.69) |
| Abdominal pain | 2908 | 3.27 | (3.16, 3.39) | 2480 | 3.81 | (3.67, 3.96) | 1328 | 3.57 | (3.38, 3.76) |
| Change in bowel habit | 778 | 5.19 | (4.84, 5.55) | 799 | 5.98 | (5.58, 6.38) | 433 | 5.75 | (5.22, 6.27) |
| Dyspepsia | 1429 | 2.33 | (2.21, 2.45) | 1297 | 2.80 | (2.65, 2.95) | 715 | 2.92 | (2.71, 3.13) |
| Dysphagia | 374 | 3.87 | (3.49, 4.26) | 406 | 4.18 | (3.78, 4.57) | 324 | 3.62 | (3.23, 4.00) |
| Rectal bleeding | 1308 | 6.00 | (5.68, 6.31) | 1279 | 7.10 | (6.72, 7.48) | 884 | 6.73 | (6.30, 7.16) |
