# Supplementary material for: Predictive values for different cancers and inflammatory bowel disease of 6 common abdominal symptoms among more than 1.9 million primary care patients in the UK: A cohort study
Source: PLoS Med. 2021 Aug 2;18(8):e1003708. doi: 10.1371/journal.pmed.1003708 (PMC8367005; doi:10.1371/journal.pmed.1003708)
Supplement: S3 Table — PPV, positive predictive value. (DOCX) [file pmed.1003708.s005.docx]

**Supplementary Table S3. Numbers of incident cases and positive predictive values (%) for cancer, by cancer site (specific to colon, rectal, esophageal and other cancers), within one year of symptom, per type of symptom, stratified by age group.**

1. **Colon cancer**

| **Sex**  Symptom | **n** | **PPV** | **(95% CI)** | **n** | **PPV** | **(95% CI)** | **n** | **PPV** | **(95% CI)** |
| --- | --- | --- | --- | --- | --- | --- | --- | --- | --- |
| **Men** | **Age 30-39** |  |  | **Age 40-49** |  |  | **Age 50-59** |  |  |
| Abdominal bloating/ distension | <5 | <0.14 | . | 7 | 0.13 | (0.03, 0.22) | 19 | 0.32 | (0.18, 0.46) |
| Abdominal pain | 24 | 0.04 | (0.02, 0.06) | 110 | 0.15 | (0.12, 0.18) | 295 | 0.43 | (0.38, 0.48) |
| Change in bowel habit | <5 | <0.17 | . | 26 | 0.42 | (0.26, 0.59) | 92 | 0.95 | (0.75, 1.14) |
| Dyspepsia | <5 | <0.01 | . | 21 | 0.05 | (0.03, 0.07) | 63 | 0.14 | (0.10, 0.17) |
| Dysphagia | <5 | <0.16 | . | <5 | <0.09 | . | <5 | <0.07 | . |
| Rectal bleeding | 9 | 0.05 | (0.02, 0.08) | 56 | 0.22 | (0.16, 0.28) | 209 | 0.84 | (0.73, 0.95) |
|  | **Age 60-69** |  |  | **Age 70-79** |  |  | **Age 80+** |  |  |
| Abdominal bloating/ distension | 38 | 0.62 | (0.42, 0.82) | 40 | 0.92 | (0.64, 1.20) | 21 | 1.14 | (0.65, 1.62) |
| Abdominal pain | 517 | 0.82 | (0.75, 0.89) | 519 | 1.16 | (1.06, 1.26) | 268 | 1.33 | (1.17, 1.49) |
| Change in bowel habit | 248 | 2.22 | (1.94, 2.49) | 296 | 2.92 | (2.60, 3.25) | 149 | 2.89 | (2.43, 3.34) |
| Dyspepsia | 131 | 0.28 | (0.23, 0.33) | 129 | 0.40 | (0.33, 0.47) | 76 | 0.58 | (0.45, 0.70) |
| Dysphagia | 20 | 0.23 | (0.13, 0.33) | 15 | 0.19 | (0.09, 0.29) | 14 | 0.27 | (0.13, 0.42) |
| Rectal bleeding | 386 | 1.72 | (1.55, 1.89) | 435 | 2.64 | (2.40, 2.89) | 244 | 3.19 | (2.79, 3.58) |
| **Women** | **Age 30-39** |  |  | **Age 40-49** |  |  | **Age 50-59** |  |  |
| Abdominal bloating/ distension | <5 | <0.04 | . | 9 | 0.05 | (0.02, 0.08) | 25 | 0.16 | (0.10, 0.23) |
| Abdominal pain | 27 | 0.02 | (0.01, 0.03) | 119 | 0.09 | (0.08, 0.11) | 292 | 0.28 | (0.25, 0.31) |
| Change in bowel habit | <5 | <0.13 | . | 27 | 0.32 | (0.20, 0.44) | 86 | 0.64 | (0.51, 0.78) |
| Dyspepsia | <5 | <0.01 | . | 15 | 0.03 | (0.01, 0.04) | 67 | 0.11 | (0.08, 0.13) |
| Dysphagia | <5 | <0.13 | . | <5 | <0.07 | . | <5 | <0.05 | . |
| Rectal bleeding | 14 | 0.07 | (0.03, 0.11) | 50 | 0.23 | (0.16, 0.29) | 189 | 0.79 | (0.68, 0.90) |
|  | **Age 60-69** |  |  | **Age 70-79** |  |  | **Age 80+** |  |  |
| Abdominal bloating/ distension | 27 | 0.23 | (0.14, 0.31) | 31 | 0.34 | (0.22, 0.46) | 32 | 0.66 | (0.43, 0.88) |
| Abdominal pain | 412 | 0.46 | (0.42, 0.51) | 515 | 0.79 | (0.72, 0.86) | 286 | 0.77 | (0.68, 0.86) |
| Change in bowel habit | 129 | 0.86 | (0.71, 1.01) | 199 | 1.49 | (1.28, 1.69) | 116 | 1.54 | (1.26, 1.82) |
| Dyspepsia | 83 | 0.14 | (0.11, 0.16) | 121 | 0.26 | (0.21, 0.31) | 105 | 0.43 | (0.35, 0.51) |
| Dysphagia | 8 | 0.08 | (0.03, 0.14) | 18 | 0.19 | (0.10, 0.27) | 26 | 0.29 | (0.18, 0.40) |
| Rectal bleeding | 258 | 1.18 | (1.04, 1.33) | 329 | 1.83 | (1.63, 2.02) | 237 | 1.80 | (1.58, 2.03) |

Counts <5 are not presented, in compliance with reporting standards for minimising disclosivity risk. Numbers are presented to two decimal places unless value <0.001, in which case the value is presented to the first significant figure.

1. **Rectal cancer**

| **Sex**  Symptom | **n** | **PPV** | **(95% CI)** | **n** | **PPV** | **(95% CI)** | **n** | **PPV** | **(95% CI)** |
| --- | --- | --- | --- | --- | --- | --- | --- | --- | --- |
| **Men** | **Age 30-39** |  |  | **Age 40-49** |  |  | **Age 50-59** |  |  |
| Abdominal bloating/ distension | <5 | <0.04 | . | <5 | <0.09 | . | <5 | <0.08 | . |
| Abdominal pain | 7 | 0.01 | (0.00, 0.02) | 23 | 0.03 | (0.02, 0.05) | 66 | 0.10 | (0.07, 0.12) |
| Change in bowel habit | 5 | 0.17 | (0.02, 0.32) | 25 | 0.41 | (0.25, 0.57) | 135 | 1.39 | (1.16, 1.62) |
| Dyspepsia | <5 | <0.01 | . | <5 | <0.01 | . | 17 | 0.04 | (0.02, 0.05) |
| Dysphagia | <5 | <0.13 | . | <5 | <0.09 | . | <5 | <0.07 | . |
| Rectal bleeding | 16 | 0.08 | (0.04, 0.12) | 82 | 0.32 | (0.25, 0.39) | 277 | 1.11 | (0.98, 1.24) |
|  | **Age 60-69** |  |  | **Age 70-79** |  |  | **Age 80+** |  |  |
| Abdominal bloating/ distension | 7 | 0.11 | (0.03, 0.20) | 9 | 0.21 | (0.07, 0.34) | 5 | 0.27 | (0.03, 0.51) |
| Abdominal pain | 91 | 0.14 | (0.11, 0.17) | 90 | 0.20 | (0.16, 0.24) | 40 | 0.20 | (0.14, 0.26) |
| Change in bowel habit | 241 | 2.15 | (1.88, 2.42) | 312 | 3.08 | (2.74, 3.42) | 116 | 2.25 | (1.84, 2.65) |
| Dyspepsia | 34 | 0.07 | (0.05, 0.10) | 50 | 0.15 | (0.11, 0.20) | 16 | 0.12 | (0.06, 0.18) |
| Dysphagia | 5 | 0.06 | (0.01, 0.11) | 8 | 0.10 | (0.03, 0.17) | <5 | <0.10 | . |
| Rectal bleeding | 430 | 1.92 | (1.74, 2.10) | 433 | 2.63 | (2.39, 2.88) | 215 | 2.81 | (2.44, 3.18) |
| **Women** | **Age 30-39** |  |  | **Age 40-49** |  |  | **Age 50-59** |  |  |
| Abdominal bloating/ distension | <5 | <0.04 | . | <5 | <0.03 | . | 7 | 0.05 | (0.01, 0.08) |
| Abdominal pain | 8 | 0.01 | (0.00, 0.01) | 23 | 0.02 | (0.01, 0.03) | 43 | 0.04 | (0.03, 0.05) |
| Change in bowel habit | <5 | <0.13 | . | 22 | 0.26 | (0.15, 0.37) | 73 | 0.55 | (0.42, 0.67) |
| Dyspepsia | <5 | <0.01 | . | <5 | <0.01 | . | 14 | 0.02 | (0.01, 0.03) |
| Dysphagia | <5 | <0.13 | . | <5 | <0.07 | . | <5 | <0.05 | . |
| Rectal bleeding | 18 | 0.09 | (0.05, 0.14) | 68 | 0.31 | (0.23, 0.38) | 200 | 0.83 | (0.72, 0.95) |
|  | **Age 60-69** |  |  | **Age 70-79** |  |  | **Age 80+** |  |  |
| Abdominal bloating/ distension | <5 | <0.04 | . | 5 | 0.06 | (0.01, 0.10) | 6 | 0.12 | (0.02, 0.22) |
| Abdominal pain | 69 | 0.08 | (0.06, 0.10) | 70 | 0.11 | (0.08, 0.13) | 55 | 0.15 | (0.11, 0.19) |
| Change in bowel habit | 81 | 0.54 | (0.42, 0.66) | 112 | 0.84 | (0.68, 0.99) | 71 | 0.94 | (0.72, 1.16) |
| Dyspepsia | 25 | 0.04 | (0.02, 0.06) | 34 | 0.07 | (0.05, 0.10) | 18 | 0.07 | (0.04, 0.11) |
| Dysphagia | <5 | <0.05 | . | 11 | 0.11 | (0.05, 0.18) | <5 | <0.06 | . |
| Rectal bleeding | 264 | 1.21 | (1.07, 1.36) | 319 | 1.77 | (1.58, 1.96) | 219 | 1.67 | (1.45, 1.89) |

Counts <5 are not presented, in compliance with reporting standards for minimising disclosivity risk. Numbers are presented to two decimal places unless value <0.001, in which case the value is presented to the first significant figure.

1. **Esophageal cancer**

| **Sex**  Symptom | **n** | **PPV** | **(95% CI)** | **n** | **PPV** | **(95% CI)** | **n** | **PPV** | **(95% CI)** |
| --- | --- | --- | --- | --- | --- | --- | --- | --- | --- |
| **Men** | **Age 30-39** |  |  | **Age 40-49** |  |  | **Age 50-59** |  |  |
| Abdominal bloating/ distension | <5 | <0.04 | . | <5 | <0.09 | . | <5 | <0.08 | . |
| Abdominal pain | <5 | <0.003 | . | 20 | 0.03 | (0.02, 0.04) | 59 | 0.09 | (0.06, 0.11) |
| Change in bowel habit | <5 | <0.13 | . | <5 | <0.08 | . | <5 | <0.05 | . |
| Dyspepsia | <5 | <0.01 | . | 28 | 0.06 | (0.04, 0.08) | 81 | 0.18 | (0.14, 0.21) |
| Dysphagia | 7 | 0.23 | (0.06, 0.40) | 50 | 0.91 | (0.66, 1.16) | 175 | 2.42 | (2.07, 2.78) |
| Rectal bleeding | <5 | <0.03 | . | <5 | <0.02 | (0.00, 0.02) | 13 | 0.05 | (0.02, 0.08) |
|  | **Age 60-69** |  |  | **Age 70-79** |  |  | **Age 80+** |  |  |
| Abdominal bloating/ distension | 10 | 0.16 | (0.06, 0.26) | <5 | <0.12 | . | <5 | <0.29 | . |
| Abdominal pain | 104 | 0.16 | (0.13, 0.20) | 94 | 0.21 | (0.17, 0.25) | 40 | 0.20 | (0.14, 0.26) |
| Change in bowel habit | <5 | <0.04 | . | 7 | 0.07 | (0.02, 0.12) | 9 | 0.17 | (0.06, 0.29) |
| Dyspepsia | 226 | 0.48 | (0.42, 0.55) | 155 | 0.48 | (0.40, 0.55) | 62 | 0.47 | (0.35, 0.59) |
| Dysphagia | 375 | 4.30 | (3.88, 4.73) | 304 | 3.88 | (3.45, 4.30) | 113 | 2.21 | (1.81, 2.62) |
| Rectal bleeding | 20 | 0.09 | (0.05, 0.13) | 19 | 0.12 | (0.06, 0.17) | 10 | 0.13 | (0.05, 0.21) |
| **Women** | **Age 30-39** |  |  | **Age 40-49** |  |  | **Age 50-59** |  |  |
| Abdominal bloating/ distension | <5 | <0.04 | . | <5 | <0.03 | . | 5 | 0.03 | (0.00, 0.06) |
| Abdominal pain | <5 | <0.003 | . | 9 | 0.01 | (0.00, 0.01) | 29 | 0.03 | (0.02, 0.04) |
| Change in bowel habit | <5 | <0.13 | . | <5 | <0.06 | . | <5 | 0.03733015 | . |
| Dyspepsia | <5 | <0.01 | . | 8 | 0.01 | (0.00, 0.02) | 40 | 0.06 | (0.04, 0.08) |
| Dysphagia | <5 | <0.13 | . | 18 | 0.25 | (0.13, 0.36) | 69 | 0.74 | (0.57, 0.92) |
| Rectal bleeding | <5 | <0.03 | . | <5 | <0.02 | . | <5 | <0.02 | . |
|  | **Age 60-69** |  |  | **Age 70-79** |  |  | **Age 80+** |  |  |
| Abdominal bloating/ distension | <5 | <0.04 | . | <5 | <0.06 | . | <5 | <0.10 | . |
| Abdominal pain | 57 | 0.06 | (0.05, 0.08) | 41 | 0.06 | (0.04, 0.08) | 25 | 0.07 | (0.04, 0.09) |
| Change in bowel habit | <5 | <0.03 | . | <5 | <0.04 | . | <5 | <0.07 | . |
| Dyspepsia | 79 | 0.13 | (0.10, 0.16) | 69 | 0.15 | (0.11, 0.18) | 44 | 0.18 | (0.13, 0.23) |
| Dysphagia | 164 | 1.70 | (1.44, 1.96) | 162 | 1.67 | (1.41, 1.92) | 101 | 1.13 | (0.91, 1.35) |
| Rectal bleeding | 6 | 0.03 | (0.01, 0.05) | 6 | 0.03 | (0.01, 0.06) | 5 | 0.04 | (0.00, 0.07) |

Counts <5 are not presented, in compliance with reporting standards for minimising disclosivity risk. Numbers are presented to two decimal places unless value <0.001, in which case the value is presented to the first significant figure.

1. **Other (bladder, breast, cervical, laryngeal, thyroid, melanoma, myeloma, prostate, testicular, vulval, vaginal)**

| **Sex**  Symptom | **n** | **PPV** | **(95% CI)** | **n** | **PPV** | **(95% CI)** | **n** | **PPV** | **(95% CI)** |
| --- | --- | --- | --- | --- | --- | --- | --- | --- | --- |
| **Men** | **Age 30-39** |  |  | **Age 40-49** |  |  | **Age 50-59** |  |  |
| Abdominal bloating/ distension | <5 | <0.04 | . | <5 | <0.09 | . | 26 | 0.44 | (0.27, 0.61) |
| Abdominal pain | 33 | 0.05 | (0.04, 0.07) | 63 | 0.09 | (0.07, 0.11) | 289 | 0.42 | (0.37, 0.47) |
| Change in bowel habit | <5 | <0.13 | . | 6 | 0.10 | (0.02, 0.18) | 38 | 0.39 | (0.27, 0.52) |
| Dyspepsia | 12 | 0.04 | (0.02, 0.06) | 32 | 0.07 | (0.05, 0.09) | 141 | 0.31 | (0.26, 0.36) |
| Dysphagia | <5 | <0.13 | . | 5 | 0.09 | (0.01, 0.17) | 35 | 0.48 | (0.32, 0.65) |
| Rectal bleeding | 7 | 0.04 | (0.01, 0.06) | 30 | 0.12 | (0.08, 0.16) | 110 | 0.44 | (0.36, 0.52) |
|  | **Age 60-69** |  |  | **Age 70-79** |  |  | **Age 80+** |  |  |
| Abdominal bloating/ distension | 69 | 1.12 | (0.86, 1.39) | 79 | 1.82 | (1.42, 2.22) | 35 | 1.89 | (1.27, 2.52) |
| Abdominal pain | 746 | 1.18 | (1.10, 1.27) | 941 | 2.10 | (1.97, 2.23) | 461 | 2.29 | (2.09, 2.50) |
| Change in bowel habit | 106 | 0.95 | (0.77, 1.13) | 165 | 1.63 | (1.38, 1.88) | 94 | 1.82 | (1.46, 2.19) |
| Dyspepsia | 413 | 0.88 | (0.80, 0.97) | 533 | 1.65 | (1.51, 1.79) | 247 | 1.87 | (1.64, 2.10) |
| Dysphagia | 110 | 1.26 | (1.03, 1.50) | 129 | 1.64 | (1.36, 1.93) | 80 | 1.57 | (1.23, 1.91) |
| Rectal bleeding | 246 | 1.10 | (0.96, 1.23) | 317 | 1.93 | (1.72, 2.14) | 149 | 1.95 | (1.64, 2.26) |
| **Women** | **Age 30-39** |  |  | **Age 40-49** |  |  | **Age 50-59** |  |  |
| Abdominal bloating/ distension | 13 | 0.09 | (0.04, 0.14) | 47 | 0.25 | (0.18, 0.33) | 85 | 0.71 | (0.56, 0.87) |
| Abdominal pain | 143 | 0.10 | (0.09, 0.12) | 344 | 0.27 | (0.24, 0.30) | 708 | 0.80 | (0.74, 0.86) |
| Change in bowel habit | 6 | 0.16 | (0.03, 0.29) | 31 | 0.37 | (0.24, 0.50) | 102 | 0.68 | (0.55, 0.81) |
| Dyspepsia | 49 | 0.11 | (0.08, 0.14) | 176 | 0.29 | (0.25, 0.34) | 423 | 0.69 | (0.62, 0.76) |
| Dysphagia | 6 | 0.15 | (0.03, 0.28) | 18 | 0.25 | (0.13, 0.36) | 89 | 0.92 | (0.73, 1.11) |
| Rectal bleeding | 18 | 0.09 | (0.05, 0.14) | 66 | 0.30 | (0.23, 0.37) | 157 | 0.72 | (0.61, 0.83) |
|  | **Age 60-69** |  |  | **Age 70-79** |  |  | **Age 80+** |  |  |
| Abdominal bloating/ distension | 85 | 0.71 | (0.56, 0.87) | 82 | 0.91 | (0.71, 1.11) | 36 | 0.74 | (0.50, 0.98) |
| Abdominal pain | 708 | 0.80 | (0.74, 0.86) | 553 | 0.85 | (0.78, 0.92) | 301 | 0.81 | (0.72, 0.90) |
| Change in bowel habit | 102 | 0.68 | (0.55, 0.81) | 98 | 0.73 | (0.59, 0.88) | 64 | 0.85 | (0.64, 1.06) |
| Dyspepsia | 423 | 0.69 | (0.62, 0.76) | 369 | 0.80 | (0.71, 0.88) | 170 | 0.69 | (0.59, 0.80) |
| Dysphagia | 89 | 0.92 | (0.73, 1.11) | 91 | 0.94 | (0.74, 1.13) | 71 | 0.79 | (0.61, 0.98) |
| Rectal bleeding | 157 | 0.72 | (0.61, 0.83) | 132 | 0.73 | (0.61, 0.86) | 105 | 0.80 | (0.65, 0.95) |

Counts <5 are not presented, in compliance with reporting standards for minimising disclosivity risk. Numbers are presented to two decimal places unless value <0.001, in which case the value is presented to the first significant figure.
