# Supplementary material for: Predictive values for different cancers and inflammatory bowel disease of 6 common abdominal symptoms among more than 1.9 million primary care patients in the UK: A cohort study
Source: PLoS Med. 2021 Aug 2;18(8):e1003708. doi: 10.1371/journal.pmed.1003708 (PMC8367005; doi:10.1371/journal.pmed.1003708)

**Supplementary Figure S1. The relative distribution of different cancer sites (among cases diagnosed with cancer) for each abdominal symptom cohort.** Note, for example, that colon and rectal cancer ‘dominate’ the cancer cases diagnosed after presenting with change in bowel habit or rectal bleeding, whereas a similarly dominant pattern is apparent for oesophageal cancer among cancer patients diagnosed after presenting with dysphagia. Nonetheless, ‘other’ cancers make up a substantial minority of cancer cases diagnosed.


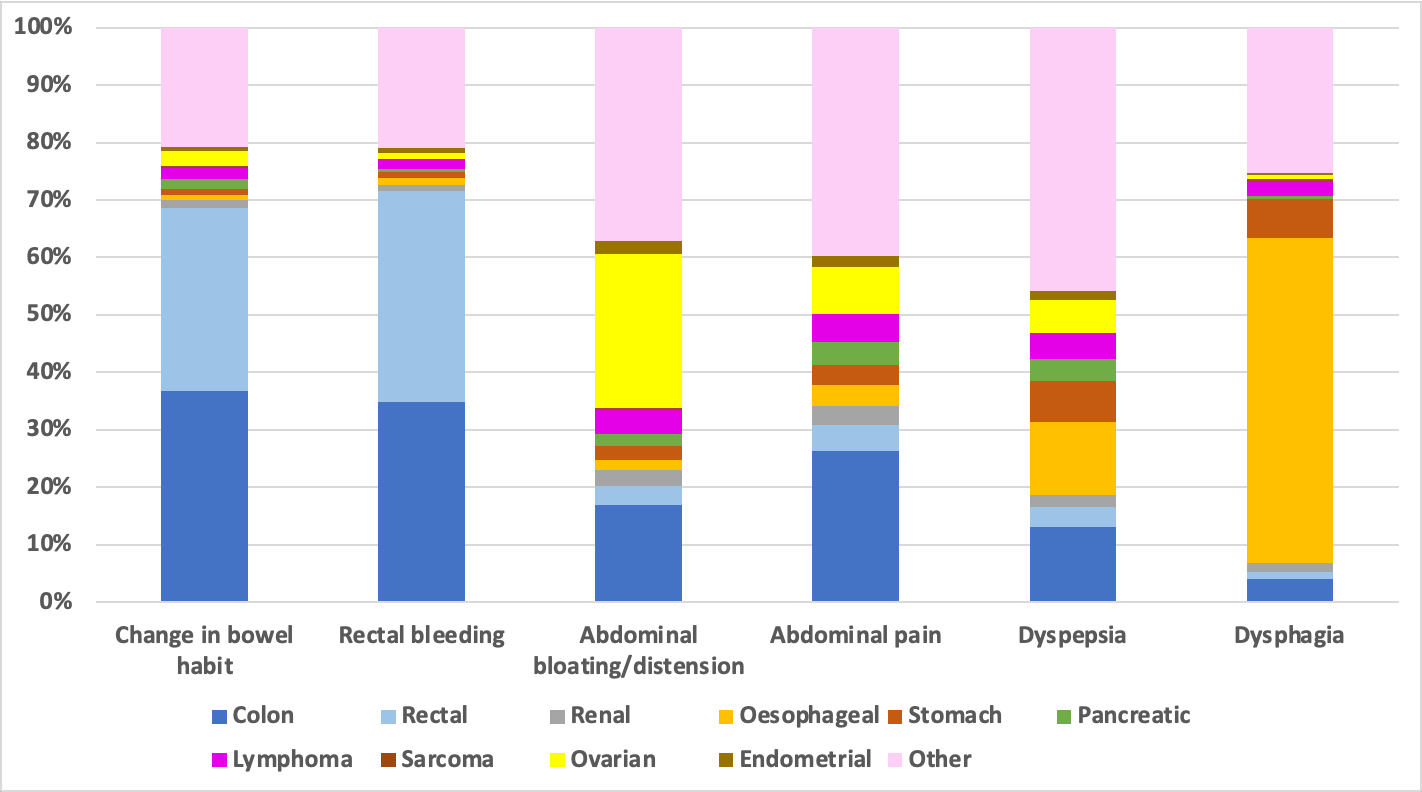

Supplement: S1 Fig — (DOCX) [file pmed.1003708.s006.docx]
