# Supplementary material for: Predictive values for different cancers and inflammatory bowel disease of 6 common abdominal symptoms among more than 1.9 million primary care patients in the UK: A cohort study
Source: PLoS Med. 2021 Aug 2;18(8):e1003708. doi: 10.1371/journal.pmed.1003708 (PMC8367005; doi:10.1371/journal.pmed.1003708)
Supplement: S2 Fig — IBD, inflammatory bowel disease. (DOCX) [file pmed.1003708.s007.docx]

**Supplementary Figure S2. The relative distribution of any cancer diagnosis or IBD among for each abdominal symptom cohort.** It can be seen that IBD contributed between 41% and 48% of all patients diagnosed with either cancer or IBD for the 5 symptoms other than dysphagia, for which IBD was diagnosed in 21%.
