# Supplementary material for: Predictive values for different cancers and inflammatory bowel disease of 6 common abdominal symptoms among more than 1.9 million primary care patients in the UK: A cohort study
Source: PLoS Med. 2021 Aug 2;18(8):e1003708. doi: 10.1371/journal.pmed.1003708 (PMC8367005; doi:10.1371/journal.pmed.1003708)
Supplement: S1 Diagram — * No records during 2000–2016 where the patient was 30–99 years old, where patient has been registered with the practice for at least 6 months and practice has achieved current recording quality standards, and 1 year before any transfer or date when the practice last provided data to THIN. THIN, The Health Improvement Network. (PDF) [file pmed.1003708.s008.pdf]

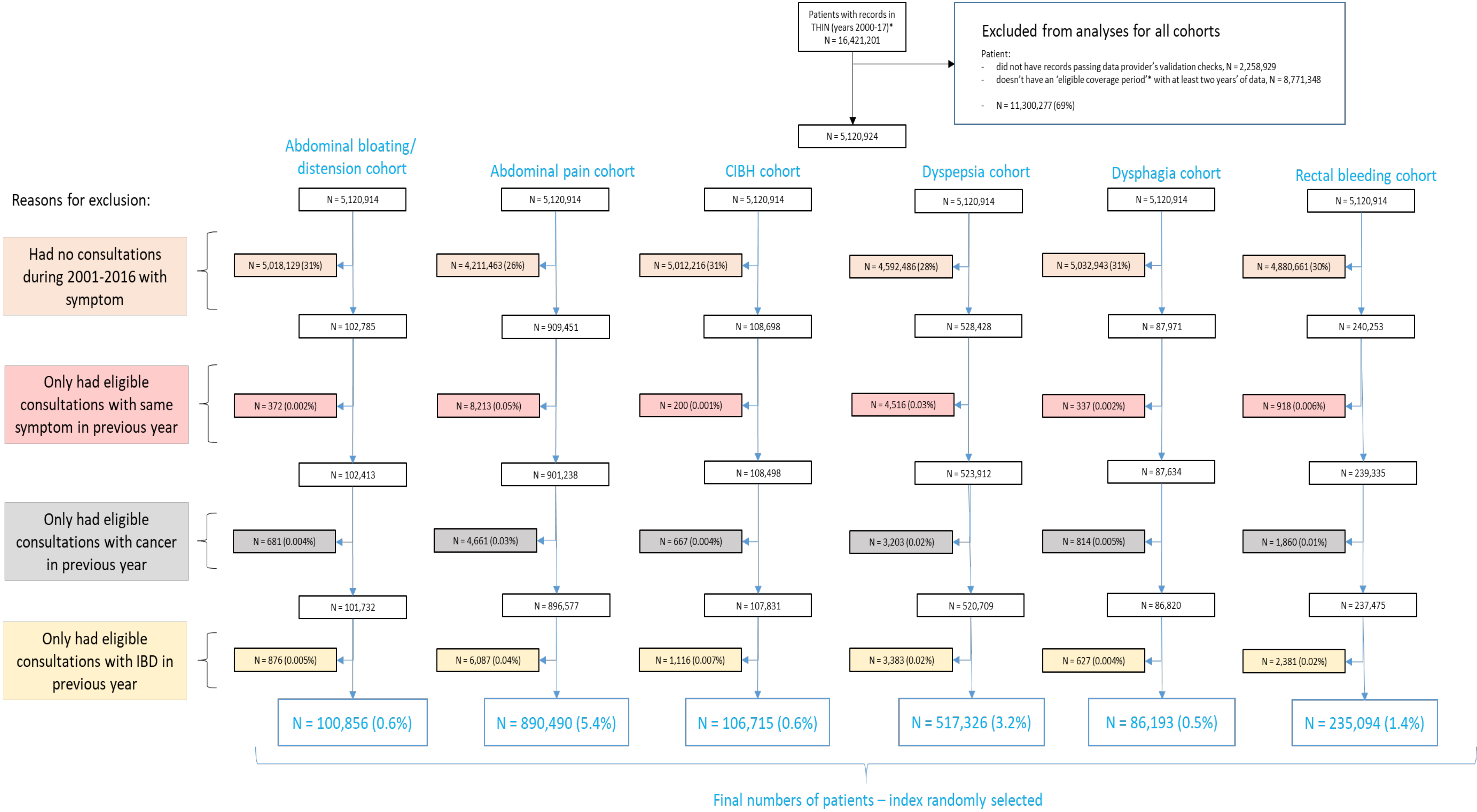

Patients with records in THIN (years 2000-17)* N = 16,421,201
Excluded from analyses for all cohorts
Patient:
- did not have records passing data provider's validation checks, N = 2,258,929
- doesn't have an 'eligible coverage period'* with at least two years' of data, N = 8,771,348
- N = 11,300,277 (69%)
N = 5,120,924
Abdominal bloating/ distension cohort
Abdominal pain cohort
CIBH cohort
Dyspepsia cohort
Dysphagia cohort
Rectal bleeding cohort
Reasons for exclusion:
N = 5,120,914
N = 5,120,914
N = 5,120,914
N = 5,120,914
N = 5,120,914
N = 5,120,914
Had no consultations during 2001-2016 with symptom
N = 5,018,129 (31%)
N = 4,211,463 (26%)
N = 5,012,216 (31%)
N = 4,592,486 (28%)
N = 5,032,943 (31%)
N = 4,880,661 (30%)
N = 102,785
N = 909,451
N = 108,698
N = 528,428
N = 87,971
N = 240,253
Only had eligible consultations with same symptom in previous year
N = 372 (0.002%)
N = 8,213 (0.05%)
N = 200 (0.001%)
N = 4,516 (0.03%)
N = 337 (0.002%)
N = 918 (0.006%)
N = 102,413
N = 901,238
N = 108,498
N = 523,912
N = 87,634
N = 239,335
Only had eligible consultations with cancer in previous year
N = 681 (0.004%)
N = 4,661 (0.03%)
N = 667 (0.004%)
N = 3,203 (0.02%)
N = 814 (0.005%)
N = 1,860 (0.01%)
N = 101,732
N = 896,577
N = 107,831
N = 520,709
N = 86,820
N = 237,475
Only had eligible consultations with IBD in previous year
N = 876 (0.005%)
N = 6,087 (0.04%)
N = 1,116 (0.007%)
N = 3,383 (0.02%)
N = 627 (0.004%)
N = 2,381 (0.02%)
N = 100,856 (0.6%)
N = 890,490 (5.4%)
N = 106,715 (0.6%)
N = 517,326 (3.2%)
N = 86,193 (0.5%)
N = 235,094 (1.4%)
Final numbers of patients – index randomly selected
